# Enhancement of Energy Transfer Efficiency with Structural Control of Multichromophore Light‐Harvesting Assembly

**DOI:** 10.1002/advs.202001623

**Published:** 2020-08-19

**Authors:** Inhwan Oh, Hosoowi Lee, Tae Wu Kim, Chang Woo Kim, Sunhong Jun, Changwon Kim, Eun Hyuk Choi, Young Min Rhee, Jeongho Kim, Woo‐Dong Jang, Hyotcherl Ihee

**Affiliations:** ^1^ Department of Chemistry Korea Advanced Institute of Science and Technology (KAIST) Daejeon 34141 Republic of Korea; ^2^ Center for Nanomaterials and Chemical Reactions Institute for Basic Science (IBS) Daejeon 34141 Republic of Korea; ^3^ KI for the BioCentury Korea Advanced Institute of Science and Technology (KAIST) Daejeon 34141 Republic of Korea; ^4^ Department of Chemistry College of Science Yonsei University Seoul 120‐749 Republic of Korea; ^5^ Department of Chemistry Inha University Incheon 22212 Republic of Korea

**Keywords:** energy transfer, Förster resonance energy transfer (FRET), light harvesting, multichromophore systems

## Abstract

Multichromophore systems (MCSs) are envisioned as building blocks of molecular optoelectronic devices. While it is important to understand the characteristics of energy transfer in MCSs, the effect of multiple donors on energy transfer has not been understood completely, mainly due to the lack of a platform to investigate such an effect systematically. Here, a systematic study on how the number of donors (*n*_D_) and interchromophore distances affect the efficiency of energy transfer (*η*_FRET_) is presented. Specifically, *η*_FRET_ is calculated for a series of model MCSs using simulations, a series of multiporphyrin dendrimers with systematic variation of *n*_D_ and interdonor distances is synthesized, and *η*_FRET_s of those dendrimers using transient absorption spectroscopy are measured. The simulations predict *η*_FRET_ in the multiporphyrin dendrimers well. In particular, it is found that *η*_FRET_ is enhanced by donor‐to‐donor energy transfer only when structural heterogeneity exists in an MCS, and the relationships between the *η*_FRET_ enhancement and the structural parameters of the MCS are revealed.

## Graphical abstract

A principle for accurately predicting the efficiency of Förster resonance energy transfer (*η*_FRET_) in multichromophore systems (MCSs) is presented. Simulations on model MCSs and spectroscopic experiments on multiporphyrin dendrimers show how the number of donors and interchromophore distances affect *η*_FRET_ and reveal that structural heterogeneity plays a key role in enhancing *η*_FRET_ in MCSs.

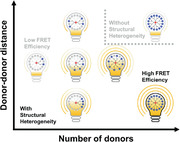

## 1 Introduction

Light‐harvesting systems consisting of multiple chromophores^[^ ^1^ ^]^ have recently attracted much interest as building blocks of molecular optoelectronic devices, for example, artificial light‐harvesting assemblies,^[^ ^2^, ^3^, ^4^, ^5^, ^6^, ^7^ ^]^ semiconductor and organic electronic materials,^[^ ^8^ ^]^ and photovoltaic solar cells.^[^ ^9^, ^10^, ^11^ ^]^ For the development and optimization of such devices, it is crucial to understand the physics and chemistry of dynamic processes, for example energy transfer, occurring in multichromophore systems (MCSs). In general, a multichromophore light‐harvesting system consists of multiple donors, which can absorb light in a wide spectral range, and multiple acceptors, which can receive the absorbed energy from the donors. For the optimum performance of such a system, it would be desirable to achieve efficient donor‐to‐acceptor (D–A) energy transfer. Energy transfer between a donor and an acceptor is often explained by the mechanism of Förster resonance energy transfer (FRET).^[^ ^12^ ^]^ According to the FRET theory, the efficiency of energy transfer is inversely proportional to the sixth power of D–A distance,^[^ ^13^, ^14^, ^15^, ^16^ ^]^ and therefore a donor–acceptor pair needs to be located within a certain distance for facilitating the transfer. While it would be ideal to achieve coherent energy transfer, it is difficult to precisely locate the donors and acceptors at positions required for coherent energy transfer to occur. In reality, incoherent energy transfer in the weak coupling regime is operational for most of MCSs.

For MCSs, however, there have been reports that the experimentally measured efficiency of excitation energy transfer (*η*_FRET_), which is defined as the probability of the acceptor to receive the energy absorbed by the donor, is much lower than the theoretically predicted efficiency.^[^ ^17^, ^18^, ^19^, ^20^, ^21^, ^22^ ^]^ It has been known that such a discrepancy in the experimental and theoretical *η*_FRET_ is mainly caused by structural heterogeneity of the molecular systems,^[^ ^22^, ^23^, ^24^, ^25^, ^26^, ^27^ ^]^ which becomes larger in complex MCSs. As an effort to compensate for the drop of *η*_FRET_ due to structural heterogeneity, many researchers have attempted to utilize donor‐to‐donor (D–D) energy transfer as a means of seeking the optimum path for efficient energy transfer toward acceptors.^[^ ^7^, ^28^ ^]^ However, the influence of D–D energy transfer on *η*_FRET_ in MCSs has been controversial. For example, Olejko and Bald^[^ ^29^ ^]^ studied the effect of the number of donors (*n*_D_) on *η*_FRET_ using the DNA origami structure as a scaffold for donors and acceptors. By varying *n*_D_ from one to four, they found that *η*_FRET_ barely changes with *n*_D_ although the amount of harvested energy increases with *n*_D_. Buckhout‐White et al.^[^ ^28^ ^]^ also investigated how *n*_D_ affects *η*_FRET_ by using the DNA structures of various shapes as a scaffold for donors and acceptor and observed only little improvement of *η*_FRET_ with the increase of *n*_D_. In contrast, Trofymchuk et al.^[^ ^7^ ^]^ and Gartzia‐Rivero et al.^[^ ^30^ ^]^ showed that, in polymeric nanoantennas composed of thousands of donors and a few acceptors, *η*_FRET_ increases with the increase of the weight ratio of donors to the mass of the polymer. Besides, there have been debates on whether the rate of D–D energy transfer influences *η*_FRET_. Vijayakumar et al.^[^ ^31^ ^]^ showed that, for self‐assemblies having different sizes of end functional groups, *η*_FRET_ increases with the increase of the rate of D–D energy transfer. In contrast, Melinger et al.^[^ ^21^ ^]^ reported that, for simple DNA dual‐rail structures containing only one or two donors, *η*_FRET_ is enhanced with the aid of D–D energy transfer but only when the rate of D–D energy transfer is faster than the rate of D–A energy transfer. Also, they showed that, as long as the D–D energy transfer is fast enough, *η*_FRET_ does not change much with the change of the rate of D–D energy transfer. Since these discrepancies were observed in studies on different MCSs, a more systematic study is called for to establish a general principle that can be used to describe the energy transfer among multiple donors and acceptors.

In this work, to better understand how the presence of multiple donors influences *η*_FRET_ in MCSs, we comprehensively investigated energy transfer in a series of model MCSs by combining simulations, chemical synthesis, and time‐resolved spectroscopy. We note that we employed the MCSs with the donors arranged in spherical symmetry, in contrast to the hybrid MCSs with donors arranged linearly as investigated in a previous study.^[^ ^32^ ^]^ In the linearly arranged donors, the D–A distance varies depending on the position of each donor. Since the D–A distance is the most critical factor that determines the FRET efficiency, we chose to investigate the spherically symmetric MCSs where the D–A distances are identical for all donors so that we can focus on the effect of homo‐FRET on FRET efficiency. As the first step of such efforts, to account for the effect of *d*_DA_, *d*_DD_, and the orientation factor (*κ* ^2^) on *η*_FRET_, we theoretically calculated *η*_FRET_ with simulations for a series of model MCSs consisting of multiple donors and a single acceptor while independently changing *n*_D_ and interchromophore distances, which include the D–A distance (*d*_DA_) and the D–D distance (*d*_DD_). Each simulation consists of Monte Carlo processes to calculate parameters needed to set up multiple differential equations, the solution of the differential equations to determine the time‐dependent excited‐state populations of donor and acceptor, and the calculation of the FRET efficiency. By running a sufficient number of simulations, we obtained *η*_FRET_ for various situations. Then, to experimentally check the validity of the calculation results of model systems, we designed and synthesized various multiporphyrin dendrimers whose *d*_DD_ and *n*_D_ were independently adjusted, and measured the dynamics and efficiencies of energy transfer in the multiporphyrin dendrimers using femtosecond transient absorption (TA) spectroscopy. From our comprehensive study, we found that *η*_FRET_ is enhanced with the aid of D–D energy transfer and such enhancement is intricately related to the structural heterogeneity of MCSs, specifically the distributions of *d*_DA_, *d*_DD_, and *κ* ^2^. The simulations presented in our study not only explain the seemingly contradicting results of previous studies but also predict *η*_FRET_ of various MCSs.

To simplify the calculation of *η*_FRET_ for an easy application to general MCSs of complicated structures, we employed the ideal dipole approximation (IDA),^[^ ^33^, ^34^ ^]^ where the electronic coupling is described as the Coulombic interaction between transition dipole moments of donor and acceptor,^[^ ^35^ ^]^ instead of the exact calculation without any approximation. While IDA has limitations compared with the exact calculation,^[^ ^36^ ^]^ for the porphyrin dendrimers investigated in our work, it turned out that the *η*_FRET_ values calculated with IDA exhibit only ≈3.5% deviation from the experimental *η*_FRET_ values.

## 2 Results

### 2.1 Calculation of *η*_FRET_ for Model Multichromophore Systems

**Figure 1:**
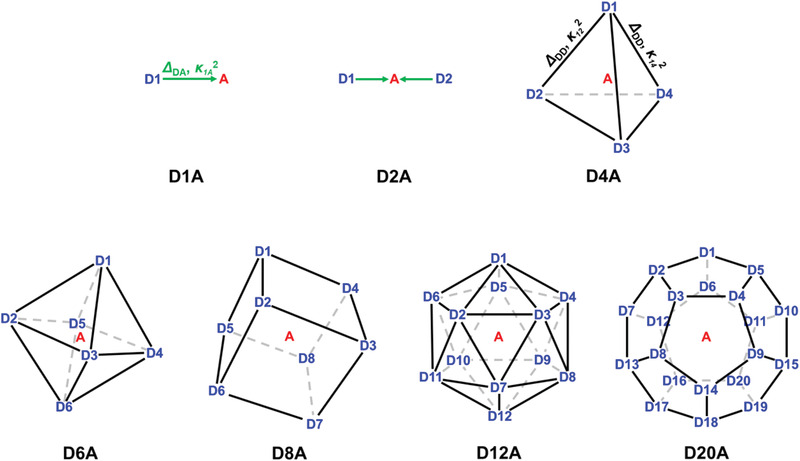
Seven model multichromophore systems used in simulations. In these model systems, the acceptor (A, red) is located at the center of multigonal structure formed by multiple donors (D, blue). The efficiency of energy transfer among chromophores is determined by the normalized interchromophore distances (*Δ*_DA_ and *Δ*_DD_) and the orientation factor (*κ* ^2^). Detailed information on the seven model systems is described in the Experimental Section.

To examine the effect of *d*_DD_, *d*_DA_, and *n*_D_ on *η*_FRET_ in MCSs, we calculated *η*_FRET_ for seven model systems that contain a single acceptor at the center and have multiple donors with various *d*_DD_, *d*_DA_, and *n*_D_ values, as shown in **Figure** **1**. In the simulations, it was assumed that only a single donor per MCS was excited and the excitation probabilities of all donors were set to be equal. To effectively compare the energy transfer behavior in these MCSs of different chemical structures and various complexities, we define the normalized interchromophore distances, *Δ*_DA_ and *Δ*_DD_, which are obtained by dividing *d*_DA_ and *d*_DD_ by the Förster radius for the D–A or D–D pair, respectively. To distinguish the donor‐to‐donor and donor‐to‐acceptor energy transfer, we denoted the D‐to‐A and the D‐to‐D energy transfer as “FRET” and “homo‐FRET,” respectively.

While MCSs tend to have inherent structural heterogeneity, the distributions of structural parameters of those systems were not considered in previous studies.^[^ ^7^, ^28^, ^29^, ^30^, ^31^, ^32^, ^37^ ^]^ Structural heterogeneity can originate from both (i) the rapid structural fluctuation of orientation factor and interchromophore distance around each of their average values and (ii) the static distribution of structural parameters such as D–A distance and orientation factor of all donors. To consider the structural heterogeneity of MCSs, we performed the simulations for calculating *η*_FRET_ in the model MCSs while considering two cases for each of *Δ*_DA_ (and *Δ*_DD_) and *κ* ^2^, which are two major parameters that govern *η*_FRET_, resulting in four cases in total. Specifically, for *Δ*_DA_ (and *Δ*_DD_), we considered either a single value or a distribution of values. A single value means that all D–A (or D–D) pairs have the same *Δ*_DA_ (or *Δ*_DD_) value, and a distribution means that those pairs can have different distance values from each other following a Gaussian distribution. For *κ* ^2^, which is defined in the Experimental Section, we checked two representative cases: the dynamic and static isotropic limit. The dynamic isotropic limit refers to a condition where the transition dipoles rotate much faster than the time scale of energy transfer and, at this limit, *κ* ^2^ has a rotationally averaged value of ⅔.^[^ ^23^ ^]^ In contrast, the static isotropic limit describes the condition where the transition dipoles rotate much slower than the time scale of energy transfer^[^ ^23^ ^]^ and thus *κ* ^2^ has a distribution of values. To summarize, as can be seen in Figure S14a,d in the Supporting Information, the following four cases were considered: i) a single value of *Δ*_DA_ (and *Δ*_DD_) and the dynamic isotropic limit, ii) a single value of *Δ*_DA_ (and *Δ*_DD_) and the static isotropic limit, iii) a finite distribution of *Δ*_DA_ (and *Δ*_DD_) and the dynamic isotropic limit, and iv) a finite distribution of *Δ*_DA_ (and *Δ*_DD_) and the static isotropic limit. Details of the simulations and the model systems are described in the Experimental Section.

**Figure 2:**
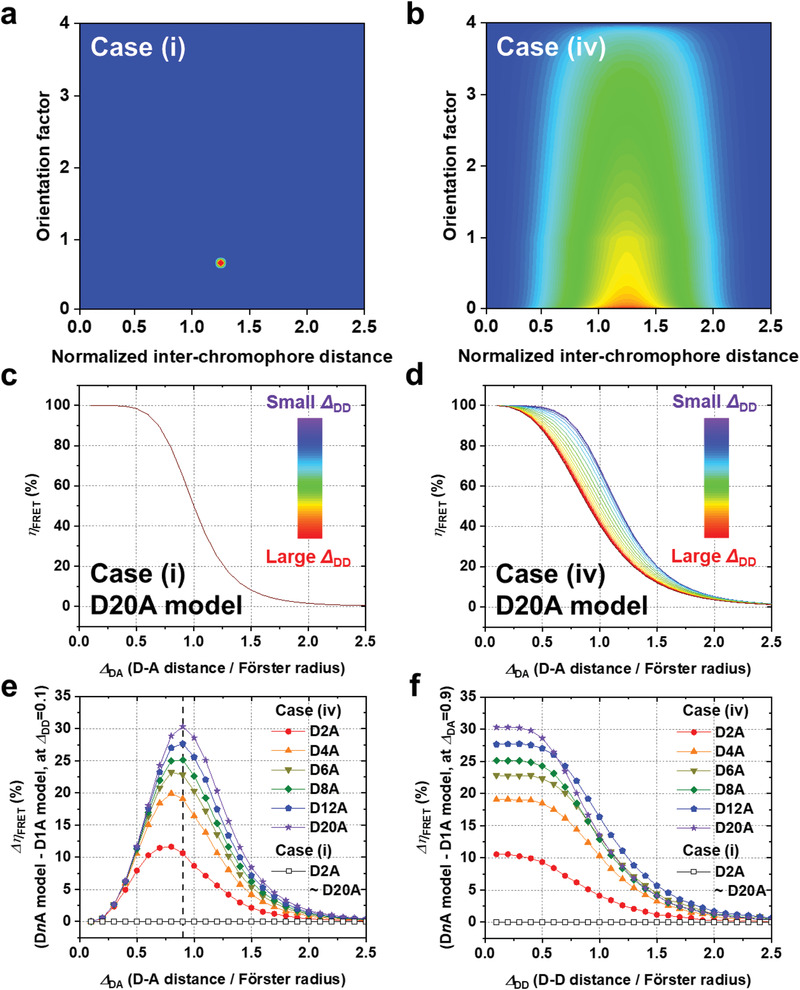
Simulation for calculating *η*_FRET_ in model MCSs. a,b) Schematic of distributions of the interchromophore distances and the orientation factor in cases (i) and (iv) used in our simulations. The distributions are shown in the form of heat maps where blue and red indicate the lowest and highest values. c,d) The FRET efficiency (*η*_FRET_) of D20A as a function of *Δ*_DA_ for various *Δ*_DD_ values. *η*_FRET_ was calculated with given values of *Δ*_DA_, *Δ*_DD_, and *κ* ^2^. *η*_FRET_ curves for various *Δ*_DD_ values are plotted following a rainbow color scheme, from red (the largest *Δ*_DD_) to purple (the smallest *Δ*_DD_). e) *Δ* *η*_FRET_ of D*n*A relative to D1A, that is *η*_FRET_(D*n*A) – *η*_FRET_(D1A), as a function of *Δ*_DA_ with the shortest *Δ*_DD_ (= 0.1) for D2A (red), D4A (orange), D6A (dark yellow), D8A (green), D12A (blue), and D20A (violet). f) *Δ* *η*_FRET_ as a function of *Δ*_DD_ with *Δ*_DA_ = 0.9, where *Δ* *η*_FRET_ is maximized for D20A. In both (e) and (f), results of case (iv) are indicated by filled symbols and those of case (i) are indicated by open symbols. All *Δ* *η*_FRET_ curves for case (i) have zero values for all *Δ*_DA_ and *Δ*_DD_ regardless of *n*_D_. The simulation results of case (ii) and case (iii) are shown in Figure S14 (Supporting Information).

The results of simulations are illustrated in **Figure** **2**; and Figure S14 (Supporting Information). Initially, the simulations were performed under the simplest condition, case (i). In particular, we calculated *η*_FRET_ while varying *Δ*_DA_ or *Δ*_DD_. As shown in Figure 2c, *η*_FRET_ increases with the decrease of *Δ*_DA_ in the D20A model, which is in good agreement with the one predicted by the Förster's equation for a single D–A pair. Here we define the degree of *η*_FRET_ enhancement induced by homo‐FRET as *Δ* *η*_FRET_ by taking the difference between *η*_FRET_ values in D*n*A model and D1A model. In case (i), as shown in Figure 2e,f, *η*_FRET_ is not enhanced at all, that is, *Δ* *η*_FRET_ = 0, with the variation of *Δ*_DA_, *Δ*_DD_, or *n*_D_. Next, the simulations were performed for the other three cases, where *Δ*_DA_ (and *Δ*_DD_) and/or *κ* ^2^ have a distribution of values. As shown in Figure 2d; and Figure S14f–h, *η*_FRET_ tends to increase with the decrease of *Δ*_DA_, in agreement with case (i). However, in contrast to case (i), *η*_FRET_ is enhanced with the variation of *Δ*_DA_ or *Δ*_DD_, that is, *Δ* *η*_FRET_ > 0, in case (ii)–(iv). Specifically, *Δ* *η*_FRET_ becomes maximal at intermediate values of *Δ*_DA_, as shown in Figure 2e; and Figure S14j–l. In addition, *Δ* *η*_FRET_ increases with the decrease of *Δ*_DD_, but such increase is saturated below a certain threshold *Δ*_DD_ value, as shown in Figure 2f; and Figure S14n,p. Especially, it should be noted that *Δ* *η*_FRET_ is positive even when *Δ*_DD_ is larger than *Δ*_DA_. In addition, *Δ* *η*_FRET_ increases with the increase of *n*_D_, even in the *Δ*_DD_ region where the increase of *Δη*_FRET_ is saturated. However, as shown in Figure 2f, *Δ* *η*_FRET_ does not simply increase with *n*_D_ when *Δ*_DD_ is larger than a certain limit. For example, *Δ* *η*_FRET_ of D20A is smaller than that of D12A at *Δ*_DD_ > 0.7, indicating that the effect of *n*_D_ on *η*_FRET_ is saturated above a certain *n*_D_ value. These results of our simulations demonstrate that the presence of distributions for *Δ*_DA_, *Δ*_DD_, and *κ* ^2^ values as in case (ii)–(iv) induces the enhancement of *η*_FRET_ (that is, *Δ* *η*_FRET_ > 0), indicating that the *Δ* *η*_FRET_ is closely related to the structural heterogeneity of MCSs. The results of the simulations are further discussed in detail in the Discussion section and the Supporting Information.

### 2.2 Design of Multiporphyrin Dendrimers

**Figure 3:**
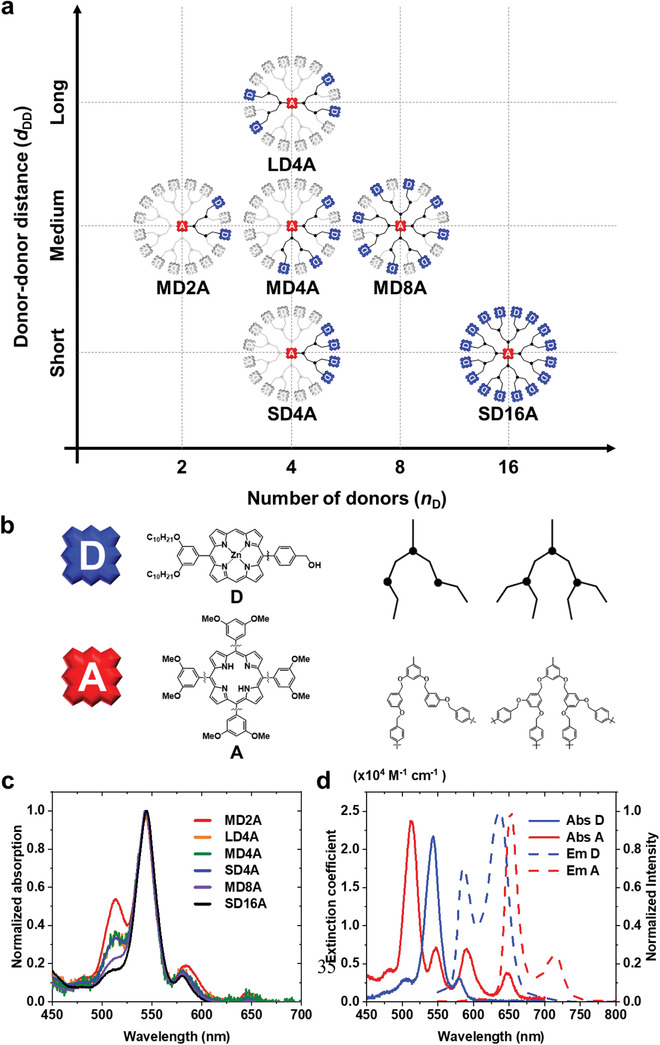
Structures of multiporphyrin dendrimers and their structural units. a) Molecular structures of multiporphyrin dendrimers used in this study represented as a function of *n*_D_ and *d*_DD_. The actual structures of the dendrimers are 3D rather than planar, but they are depicted as the planar configurations for the sake of clear presentation. In all of the six dendrimers, each zinc porphyrin donor (D, blue) is connected to a free‐base porphyrin acceptor (A, red) by the linker of the same length. For easier comparison among the dendrimers, the structures of all the dendrimers are drawn on the same frame as SD16A, and the spots without any chromophore are indicated in gray. A dendrimer containing short D–D pairs is labeled as S, a dendrimer containing long D–D pairs without any short D–D pair is labeled as L, and all other dendrimers without any short or long D–D pairs are labeled as M. b) Molecular structures of zinc porphyrin donor (D) unit, free‐base porphyrin acceptor (A) unit, and linkers connecting the D and A units in the multiporphyrin dendrimers. c) Normalized absorption spectra of multiporphyrin dendrimers. d) Absorption and emission spectra of monomeric donor and acceptor.

To experimentally test the validity of the results of simulations, we designed and synthesized the MCSs with various *n*_D_ and *d*_DD_ values. Since it is difficult to experimentally prepare the ideal forms of MCSs as the ones used for the simulations, we instead prepared multiporphyrin dendrimers as the model compounds. The multiporphyrin dendrimers should be an excellent model system because both *n*_D_ and *d*_DD_ can be adjusted independently by controlling the type, number, and position of linkers while keeping *d*_DA_ and the spectral overlap integral for D–A pairs unchanged for each donor.^[^ ^6^, ^38^ ^]^ In this context, we synthesized a total of six multiporphyrin dendrimers with various combinations of *n*_D_ and *d*_DD_ values, as illustrated in **Figure** **3**. For example, SD4A, MD4A, and LD4A have the same *n*_D_ value but different *d*_DD_ values, and MD2A, MD4A, and MD8A have the same *d*_DD_ value but different *n*_D_ values. The absorption and emission spectra of dendrimers and their constituent donor and acceptor units are shown in Figure 3c,d. The weak electronic coupling between ground‐state porphyrins in dendrimers was confirmed by the absence of the spectral broadening or shift in their spectra. The Förster radius for the D–A pair was calculated to be 2.43 nm from the static spectroscopic data, as described in the Supporting Information.

**Figure 4:**
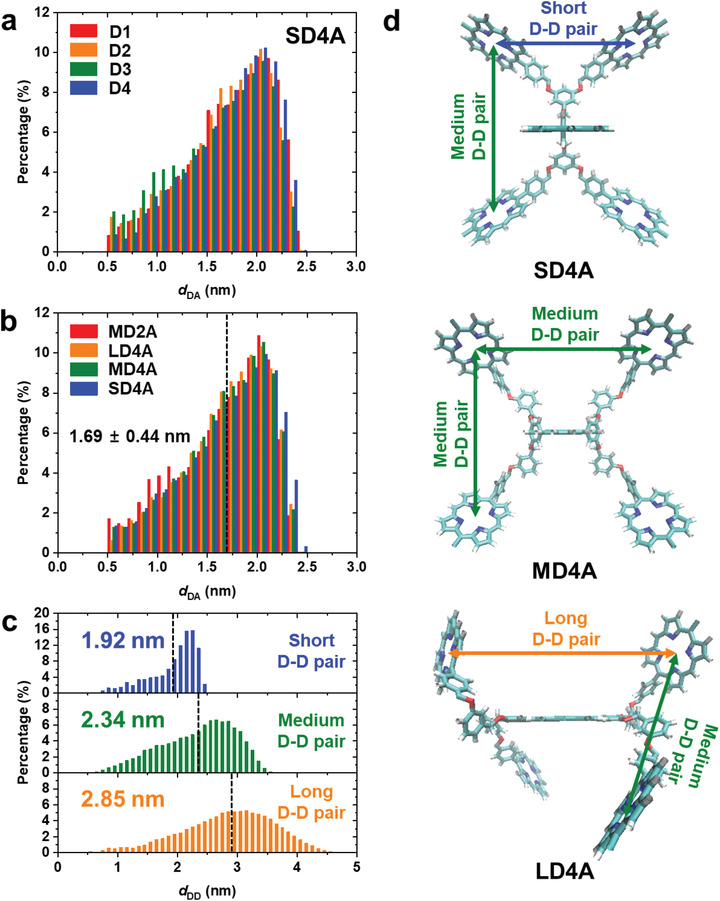
Distributions of *d*_DA_ and *d*_DD_ of multiporphyrin dendrimers obtained from MD simulations. a) Distributions of *d*_DA_ for each of the four donors in SD4A. b) Averaged distributions of *d*_DA_ for MD2A, LD4A, MD4A, and SD4A. The mean value of all the averaged *d*_DA_ distributions for the six dendrimers is 1.69 ± 0.44 nm. c) Averaged distributions of *d*_DD_ for short D–D pairs (blue), medium D–D pairs (green), and long D–D pairs (orange) in all six dendrimers. The classification into short, medium, and long D–D pairs are defined in the caption of Figure 3a. The mean value of each averaged *d*_DD_ distribution is indicated by a dashed line. Examples of short, medium, and long D–D pairs are illustrated in (d). d) Average structures of multiporphyrin dendrimers with four donors. The positions of each D–D pair are indicated. For MD4A, the distance between donors attached to different linkers from the acceptor (2.44 nm) is slightly longer than the distance between donors connected with the same linker from the acceptor (2.34 nm) although both pairs of donors are classified into medium D–D pairs.

To estimate the equilibrium structures of the dendrimers, molecular dynamics (MD) simulations were performed for the multiporphyrin dendrimers. The results of MD simulations are shown in **Figure** **4**, and details of MD simulations are described in the Supporting Information. According to the MD simulation, the distributions of *d*_DA_ are similar for all donors in a dendrimer, as shown in Figure 4a, and the averaged *d*_DA_ distributions for all donors in a dendrimer are similar for all of the dendrimers, as shown in Figure 4b. This result suggests that the mean *d*_DA_ of all dendrimers does not change regardless of the variation of *d*_DD_ and *n*_D_. Unlike *d*_DA_, however, the averages of *d*_DD_ distributions are different for each pair of donors, depending on their geometry in a dendrimer, as shown in Figure 4c. Therefore, in these dendrimers, we can clearly examine the effect of *d*_DD_ and *n*_D_ on *η*_FRET_, independent of *d*_DA_. The structural information on the porphyrin dendrimers obtained from the MD simulations was used as input for calculating the theoretical *η*_FRET_ for the dendrimers using the simulations described above.

### 2.3 FRET Efficiencies of Multiporphyrin Dendrimers

**Figure 5:**
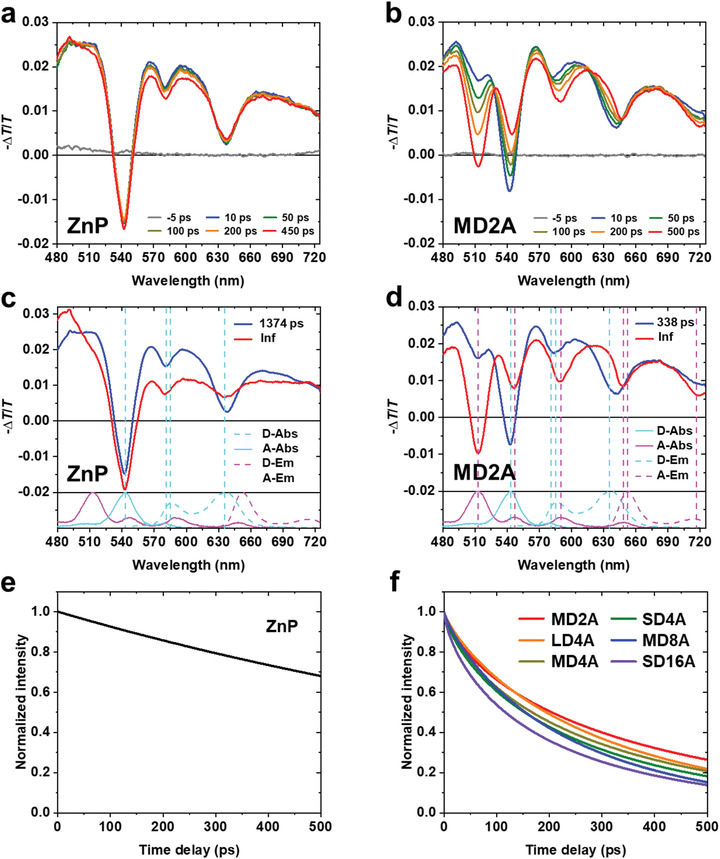
Transient absorption (TA) spectra and decay‐associated spectra (DAS) of monomeric zinc porphyrin and MD2A dendrimer. a,b) TA spectra of the monomeric zinc porphyrin (a) and MD2A (b) at various time delays. c,d) DAS of the monomeric zinc porphyrin (c) and MD2A (d) obtained from the global target analysis of TA spectra. The steady‐state absorption spectra (solid line) and emission spectra (dashed line) of the donor (cyan) and the acceptor (magenta) are shown together at the bottom. The vertical dashed lines indicate the peak positions of steady‐state absorption and emission spectra. Temporal decay of the DAS of e) monomeric zinc porphyrin and f) multiporphyrin dendrimers.

To measure the dynamics of energy transfer in the multiporphyrin dendrimers, femtosecond TA spectroscopy^[^ ^39^ ^]^ was employed, as described in the Experimental Section. The results of TA measurement for multiporphyrin dendrimers and monomeric zinc porphyrin are shown in **Figure** **5**; and Figure S10 (Supporting Information). In Figure 5, it can be seen that the TA spectra of the monomeric zinc porphyrin exhibit the decay of the 1.3 ns time constant. Considering the characteristic spectral evolution shown in decay associated spectra and that the singlet excited state of zinc porphyrin species mainly decays via intersystem crossing to a triplet state rather than fluorescence emission, we can assign the TA decay of monomeric zinc porphyrin to intersystem crossing.^[^ ^40^, ^41^, ^42^, ^43^, ^44^ ^]^ Unlike the spectral evolution of the monomeric zinc porphyrin, the TA spectra of dendrimers show distinct and fast spectral changes. The ground state bleach (GSB) of donor (543 nm) is reduced, while the GSB of acceptor (517 nm) is increased, and the SE of donor (637 nm) is shifted to longer wavelengths. Based on these features, we attribute the spectral changes of dendrimers to the FRET from donors to the acceptor.

To characterize the dynamics of the time‐dependent spectral changes in the TA spectra of various dendrimers, we globally fitted the TA spectra in the entire spectral range of our measurement. The resultant decay‐associated spectra (DAS) of the monomeric zinc porphyrin and MD2A are shown in Figure 5c,d, respectively, and the temporal decays of those DAS are shown in Figure 5e,f.^[^ ^45^ ^]^ Since there exist multiple donor–acceptor pairs in each dendrimer and *d*_DA_ has a distribution of values rather than a single value, the dendrimers are expected to exhibit complex, parallel energy transfer dynamics, as manifested in Figure 5f.^[^ ^46^ ^]^ To reflect such complicated dynamics, we fitted the decay of TA spectra using a stretched exponential function, which is suitable for describing a relaxation process that takes place in a parallel manner with varying time scales

(1) $$\begin{array}{c} I_{\mathrm{TA}}\left(t\right)\;=\;A\;\times \;e^{-{\left(t/\tau_{\mathrm{DA}}\right)}^{\beta }}+I_{\mathrm{long}} \end{array}$$

where *τ*_DA_ is the time constant of FRET process and *β* is related to the degree to which the exponential function is stretched. The fit parameters are presented in **Table** **1**, and *η*_FRET_ was calculated using Equation (4).^[^ ^47^, ^48^ ^]^ It can be seen that, for various dendrimers, *η*_FRET_ differs by up to 11%. Especially, *η*_FRET_ of MD8A is higher than those of MD4A and MD2A although these three dendrimers have identical mean values of *d*_DD_ and *d*_DA_. For the three dendrimers having four donors (SD4A, MD4A, and LD4A), it can also be seen that *η*_FRET_ of a dendrimer with shorter *d*_DD_ is larger than that of a dendrimer with longer *d*_DD_. These results show that *η*_FRET_ of a dendrimer is enhanced by the increase of *n*_D_ or the decrease of *d*_DD_.

**Table 1:** Summary of the time constants and *β* values observed in TA spectra of multiporphyrin dendrimers and calculated FRET efficiency (*η*_FRET_)

	*τ*_DA_ ^a)^ [ps]	*β* ^a)^	Experimental *η*_FRET_ ^b)^ [%]	Theoretical *η*_FRET_ ^c)^ [%]	Modified theoretical *η*_FRET_ ^d)^ [%]
MD2A	338 ± 29	0.73 ± 0.03	74.0 ± 2.2	72.1	74.1
LD4A	302 ± 4	0.83 ± 0.02	76.8 ± 0.3	74.7	76.5
MD4A	273 ± 4	0.75 ± 0.02	79.0 ± 0.3	77.0	78.6
SD4A	248 ± 18	0.76 ± 0.04	80.9 ± 1.4	78.2	79.8
MD8A	238 ± 17	0.85 ± 0.05	81.7 ± 1.3	78.8	80.4
SD16A	194 ± 15	0.72 ± 0.05	85.1 ± 1.1	83.9–85.8	85.2–87.1

a) All FRET time constants (*τ*_DA_) and *β* values were determined from the global fits of the first and second right singular vectors of TA spectra

b) Experimental *η*_FRET_s were calculated from the time constants obtained from TA measurements by using Equation (S4) (Supporting Information). We note that the decay time constant of the TA spectra of monomeric zinc porphyrins is 1.3 ns

c) Theoretical *η*_FRET_s were calculated with the simulations using the structural information of dendrimers obtained from MD simulations. We note that the mean value (1.69 nm) of the *d*_DA_ distribution in Figure 4b was used

d) Modified theoretical *η*_FRET_s were calculated with the simulations using the *d*_DA_ value of 1.65 nm, instead of 1.69 nm. The *d*_DA_ value of 1.65 nm was obtained, while we vary *d*_DA_ to match the theoretical *η*_FRET_ with the experimental value. Based on the good agreement of the modified theoretical and experimental *η*_FRET_s, we can infer that the value of 1.65 nm is the effective *d*_DA_ in the multiporphyrin dendrimers.

To verify whether the simulations accurately describe the FRET dynamics in MCSs, we predicted the theoretical *η*_FRET_ of the multiporphyrin dendrimers with the simulations using the structural information obtained from the MD simulations. Details of the calculation of theoretical *η*_FRET_ are described in the Supporting Information. For each dendrimer, the theoretical *η*_FRET_ is compared with the experimentally determined *η*_FRET_. As shown in Table 1, the theoretical *η*_FRET_s agree well with the experimental *η*_FRET_s, but overall they are underestimated by ≈3.5% compared with the experimental *η*_FRET_s. These discrepancies might be attributed to the limitation of IDA applied to the Förster's equation and the slight inaccuracy of the *d*_DA_ value obtained from MD simulations. Therefore, we varied *d*_DA_ to match the theoretical *η*_FRET_ with the experimental value and calculated the modified theoretical *η*_FRET_s. The modified theoretical and the experimental *η*_FRET_s were in agreement with each other when the *d*_DA_ value of 1.65 nm were used, instead of 1.69 nm. The excellent agreement of the modified theoretical and the experimental *η*_FRET_s implies that our simulations well describes the FRET behavior in MCSs.

## 3 Discussion

The excitation energy of donors can be transferred to the acceptor either directly or through multiple steps involving homo‐FRET among donors. If the energy transfer via these detour paths involving homo‐FRET is more efficient than the direct transfer to the acceptor, *η*_FRET_ will be enhanced compared with when homo‐FRET does not occur. From the simulations of energy transfer for the seven model systems, we comprehensively examined how various structural parameters (*n*_D_, *Δ*_DD_, *Δ*_DA_, and *κ* ^2^) of an MCS affects *η*_FRET_. In particular, we emphasize how structural heterogeneity in an MCS affects *η*_FRET_ by comparing case (i) and the other three cases.

According to previous studies, structural heterogeneity tends to deteriorate *η*_FRET_.^[^ ^22^, ^23^, ^24^, ^25^, ^26^, ^27^, ^28^ ^]^ However, as can be seen in Figure 2; and Figure S14 (Supporting Information), *η*_FRET_ can be enhanced with the aid of structural heterogeneity caused by the presence of multiple donors and homo‐FRET among donors. As shown in Figure 2f; and Figure S14n,o (Supporting Information), *η*_FRET_ is enhanced with the decrease of *Δ*_DD_ in case (ii), (iii), and (iv), implying that the increase of the homo‐FRET rate (due to the decrease of *Δ*_DD_) activates more detour FRET paths of high efficiencies. As mentioned in the simulation results, the increase of *Δ* *η*_FRET_ with the decrease of *Δ*_DD_ is saturated below a certain threshold *Δ*_DD_ value, of which the reason is discussed in Note S8 (Supporting Information). Considering that the enhancement of *η*_FRET_ is not observed in case (i), we can infer that structural heterogeneity in the MCS plays an important role in the enhancement of *η*_FRET_ induced by homo‐FRET. In case (i), *η*_FRET_ does not change at all with the variation of *n*_D_ or *Δ*_DD_, suggesting that homo‐FRET does not affect the FRET efficiency without any structural heterogeneity. In contrast, in cases (ii), (iii), and (iv) with heterogeneity of *Δ*_DA_ (and *Δ*_DD_) and/or *κ* ^2^, *Δ* *η*_FRET_ is positive and changes sensitively with the variation of *n*_D_ and *Δ*_DD_, indicating the strong influence of structural heterogeneity on the homo‐FRET‐induced enhancement of *η*_FRET_. Thus, according to our simulations, only when there exists structural heterogeneity in an MCS, homo‐FRET enhances *η*_FRET_, thus compensating for the loss of *η*_FRET_ induced by the structural heterogeneity to some extent.

While we demonstrated that structural heterogeneity is essential for the homo‐FRET‐induced enhancement of *η*_FRET_, it would be desirable to describe their relationship quantitatively. If we define the FRET efficiency, *Φ*_FRET_, of an individual donor, the structural heterogeneity in an MCS can be manifested as a distribution of *Φ*_FRET_ for multiple donors. In other words, when there exists structural heterogeneity, the distribution of *Φ*_FRET_ would have a finite width. While *η*_FRET_ is enhanced in cases (ii), (iii), and (iv), where *Φ*_FRET_ has a distribution of values, the *Δ*_DA_ dependences of *Δ* *η*_FRET_ for those three cases are different from each other, as shown in Figure 2e; and Figure S14j,k (Supporting Information), because the distribution of *Φ*_FRET_ arises from different origins in those three cases. For case (ii), the distribution of *Φ*_FRET_ is governed by only the distribution of *κ* ^2^. For case (iii), the distribution of *Φ*_FRET_ is governed by only the distribution of *Δ*_DA_. For case (iv), the distribution of *Φ*_FRET_ is governed by the distributions of both *κ* ^2^ and *Δ*_DA_. At *Δ*_DA_ > 1.5 in case (ii) and at *Δ*_DA_ < 0.4 in case (iii), *Δ* *η*_FRET_ induced by homo‐FRET is negligibly small. Such limited enhancement of *η*_FRET_ in those *Δ*_DA_ regions indicates that the distribution of *Φ*_FRET_ is too narrow to enhance *η*_FRET_. In fact, it can be seen in Figure S17 (Supporting Information) that *Δ* *η*_FRET_ is governed by the width of *Φ*_FRET_ distribution for cases (ii) and (iii). In contrast, in case (iv), *η*_FRET_ is enhanced in the entire *Δ*_DA_ region because the variation of *κ* ^2^ and the variation of *Δ*_DA_ induces the enhancement of *η*_FRET_ at small and large *Δ*_DA_ values, respectively, thus complementing each other. In other words, in case (iv), the distributions of both *κ* ^2^ and *Δ*_DA_ contribute to the enhancement of *η*_FRET_, as confirmed by Figure S18 (Supporting Information). Further discussions on the dependence of *η*_FRET_ on *Δ*_DA_ are described in Notes S7 and S8 (Supporting Information).

As shown in Figure 2e; and Figure S14j,k (Supporting Information), *η*_FRET_ is enhanced overall by the increase of *n*_D_, in the order of D2A, D4A, D6A, D8A, D12A, and D20A. Such dependence of *η*_FRET_ on *n*_D_ can be attributed the increase of donors that are available for homo‐FRET. However, it is noteworthy that the *Δ* *η*_FRET_ is not exactly proportional to *n*_D_ when *Δ*_DD_ is larger than ≈0.7, as shown in Figure 2f; and Figure S14n,o (Supporting Information). For example, at *Δ*_DD_ = 1.3, the *Δ* *η*_FRET_ increases in the order of D2A, D4A, D8A, D20A, D6A, and D12A, as shown in Figure S21 (Supporting Information). To find the origin of the deviation from the dependence on *n*_D_, we checked how the average homo‐FRET efficiency depends on *Δ*_DD_. At *Δ*_DD_ = 0.1, both the average homo‐FRET efficiencies to the nearest and the second nearest donors are close to 100%. By contrast, at *Δ*_DD_ = 1.3, the average efficiency of homo‐FRET to the nearest donors is 20%, while that to the second‐nearest donors is only 5% at best. Such a large difference in the homo‐FRET efficiencies between the nearest and the second‐nearest donors means that only homo‐FRET to the nearest donors is effective. Base on this observation, for all model systems, we checked the number of the nearest donors (*n*_ND_), which is 2, 3, 3, 3, 4, and 5 for D2A, D4A, D8A, D20A, D6A, and D12A, respectively. Indeed, the order of the model systems in terms of *n*_ND_ exactly matches the observed order of model systems in terms of *η*_FRET_, indicating that *η*_FRET_ is governed by *n*_ND_ rather than *n*_D_, the total number of donors. To further verify the influence of *n*_ND_ on *η*_FRET_, we also performed simulations for pseudomodel systems where all donors are equally spaced from each other, that is, *n*_ND_ equals *n*_D_. As shown in Figure S22 (Supporting Information), the simulation results show that the *Δ* *η*_FRET_ is proportional to *n*_D_ for such model systems. Thus, the *Δ* *η*_FRET_ is governed *n*_ND_ rather than *n*_D_. Ultimately, the 3D arrangement of chromophores and the extent of structural heterogeneity in an MCS would be important factors that determine the homo‐FRET‐induced enhancement of *η*_FRET_ because *n*_ND_ would be determined by these factors.

We can compare our simulation results with those of the previous studies, as discussed in Note S9 (Supporting Information). Notably, the simulations performed in a previous spectroscopic study on DNA‐fluorophore MCSs showed that when *d*_DD_ is increased by 54% from 13 Å (*Δ*_DD_ = 0.25) to 20 Å (*Δ*_DD_ = 0.38), *η*_FRET_ changes only by 3% and suggested that *η*_FRET_ is not much affected by the rate of homo‐FRET, as long as the homo‐FRET rate is significantly faster than the hetero‐FRET rate.^[^ ^21^ ^]^ These results are consistent with ours. Such insensitivity of *η*_FRET_ to *Δ*_DD_ should be attributed to the limited *Δ*_DD_ range (0.25–0.38) examined in that study. As can be seen in Figure 2f, our simulations show that *Δ* *η*_FRET_ is saturated in that *Δ*_DD_ range. On the other hand, based on their own simulation, they inferred that the enhancement of *η*_FRET_ induced by homo‐FRET occurs only when homo‐FRET is faster than FRET. By contrast, our simulations suggest that *η*_FRET_ can be enhanced even when *Δ*_DD_ is larger than *Δ*_DA_ (that is, homo‐FRET is slower than FRET), as can be seen in Figure 2f (*Δ* *η*_FRET_ > 0 at *Δ*_DD_ > *Δ*_DA_). This result of our simulations implies that homo‐FRET still effectively funnels the excitation energy toward the detour FRET paths of high efficiencies even when homo‐FRET is slower than FRET. In agreement with this result of our simulation, our TA measurements show that *η*_FRET_ is enhanced in the dendrimers investigated in this work, all of which have smaller *Δ*_DA_ (0.7) than *Δ*_DD_ (1.0 for SD4A and SD16A, 1.2 for MD2A, MD4A, and MD8A and 1.5 For LD4A). In a similar study on MCSs based on DNA origami structure,^[^ ^29^ ^]^ *η*_FRET_ was determined from the measured fluorescence lifetime of the donor, while *n*_D_ being varied from one to four, and it was found that *η*_FRET_ was barely affected by the increase of *n*_D_. This observation can be easily rationalized by noting that these MCSs correspond to conditions where *η*_FRET_ is hardly affected by *n*_D_. Similarly, in a study on MCSs consisting of various DNA networks,^[^ ^28^ ^]^ it was found that *η*_FRET_ does not show clear dependence on *n*_D_. Here we can interpret that such insensitivity of *η*_FRET_ with respect to *n*_D_ can be attributed to the low formation efficiency of some of the MCSs used in that study. In fact, when only the MCSs of high formation efficiencies are considered, it can be clearly seen that *η*_FRET_ is enhanced with the increase of *n*_D_. In addition, for the MCSs of high formation efficiencies, *Δ*_DA_ dependence of *Δ* *η*_FRET_ is also in good agreement with the results of our simulations. Thus, our simulations can account for the conflicts of the previous studies and serve as a tool that properly describes the *η*_FRET_ in MCSs.

We note that we used IDA for all of the simulations. One of the limitations of IDA is that it cannot account for the energy transfer between orthogonal chromophores, which should be treated by taking into account molecular vibrations and environmental fluctuations.^[^ ^49^, ^50^, ^51^, ^52^, ^53^ ^]^ The error caused by IDA generally increases with the decrease of the *d*_DA_ distance and the increase of the size of the chromophore.^[^ ^36^ ^]^ The porphyrin dendrimers investigated in our work have *d*_DA_ of 16.9 Å and the porphyrin unit has a size of ≈7 Å,^[^ ^54^, ^55^ ^]^ giving the D–A separation of ≈2.4 in molecular size unit. According to the previous work,^[^ ^36^ ^]^ with this D–A separation value, the error is estimated to be ≈7.5%, which is comparable to the ≈3.5% deviation between the *η*_FRET_ estimated using IDA and the experimentally measured value in our work. Since such an error due to IDA will apply to all porphyrin dendrimers systematically, it should not affect the relative *η*_FRET_ of the porphyrin dendrimers, which is of our interest in this work. In fact, the variation of the calculated *η*_FRET_ for different porphyrin dendrimers is in excellent agreement with the trend of the experimentally measured values for those dendrimers (see Table 1). While the correction using the exact approach instead of IDA will reduce the error, our approach using IDA requires only simple calculation and can be easily applied to MCSs of various chemical structures.

## 4 Conclusion

Based on the simulations on model MCSs for four different cases and the TA measurements on multiporphyrin dendrimers, we revealed the followings on the energy transfer efficiencies and the structural parameters of the MCSs: i) *η*_FRET_ is enhanced by homo‐FRET only when there exists structural heterogeneity in the MCS; ii) the enhancement of *η*_FRET_ is directly related to the distribution of *Φ*_FRET_, of which the width is governed by the distribution of *κ* ^2^ at small *Δ*_DA_ values and by the distribution of *Δ*_DA_ at large *Δ*_DA_ values; iii) *η*_FRET_ is enhanced by the increase of *n*_D_, especially by the increase of *n*_ND_. Based on these lessons learned from this study, we can explain why the conclusions from previous studies seemingly conflict with each other. The excellent agreement between the experimental and the calculated *η*_FRET_s (see Table 1) demonstrated for the systematically designed MCSs investigated in our study would provide a principle for accurately predicting *η*_FRET_ in various MCSs. Our findings can help a better understanding of energy transfer in MCSs, including natural light‐harvesting systems, and provide a design principle for novel optoelectronic devices.

## 5 Experimental Section

##### Calculation of *η*_FRET_ for Model Multichromophore Systems

For each model MCS, *η*_FRET_ was calculated at given *Δ*_DA_ and *Δ*_DD_ values by averaging *Φ*_FRET_, the FRET efficiency of an individual donor, obtained from 100,000 simulations, which are large enough to sample the distributions of structural parameters. Each simulation consists of the Monte Carlo processes to calculate the parameters necessary for setting up multiple differential equations to determine the time‐dependent excited‐state populations of donor and acceptor, the solution of the differential equations which provide the excited‐state populations of chromophores as a function of time at given *Δ*_DA_ and *Δ*_DD_ values, and the calculation of *Φ*_FRET_, by dividing the final excited‐state population of the acceptor by the initial excited‐state population of the donor. For the calculation of *Φ*_FRET_, it was assumed that the intrinsic decay rate of the excited‐state population of the acceptor was negligibly small. Under this condition, the excited‐state population of the acceptor was changed only by FRET from the donor. For each model MCS, such simulations were performed for all four cases discussed in the main text (cases (i)–(iv)). For cases (i) and (ii) where *Δ*_DA_ (and *Δ*_DD_) has a single value, 25 *Δ*_DA_ values and 25 *Δ*_DD_ values were used between 0.1 and 2.5 with the increment of 0.1. For cases (iii) and (iv) where *Δ*_DA_ (and *Δ*_DD_) has a finite distribution, a random number was generated for *Δ*_DA_ (and *Δ*_DD_) following its Gaussian distribution. The mean value of the Gaussian distribution was increased from 0.1 to 2.5 with an increment of 0.1. The relative standard deviation of the Gaussian distribution was set to be 0.2.

In general, MCSs are composed of various types of chromophores. For simplicity, however, only a single type of energy‐donor chromophore and a single type of energy‐acceptor chromophore were considered for seven ideal model MCSs investigated in the study. In those model systems, an acceptor is surrounded by donors that are located as far apart from each other as possible. Each donor can have a distribution of *Δ*_DA_ for cases (iii) and (iv), but the mean *Δ*_DA_s for all donors were set to be identical for all donors so that the effect of the variation of *Δ*_DA_ on *η*_FRET_ can be excluded. In addition, the mean *Δ*_DD_s between the nearest donors were also set to be identical for all pairs of the nearest donors. For simulations on model MCSs with the number of *n*_ND_ set to be the same as *n*_D_, the mean *Δ*_DD_s among all pairs of donors were set to be equal. In the simulations, the excitation probabilities of all donors were set to be equal, but the simultaneous excitation of two or more donors was not considered. The direct excitation of the acceptor was not considered, either. The unidirectional D–A energy transfer as well as the D–D energy transfer were considered.

Energy transfer among multiple chromophores of two different types cannot be described only with a single Förster's equation but can be described with a combination of multiple Förster's equations. Therefore, differential equations that describe energy transfer for a model MCS consisting of *n* donors and a single acceptor can be expressed as follows

(2) $$\begin{array}{ccc} \frac{dD_{a}\left(t\right)}{dt} & = & -\left(k_{D}+k_{D_{a}A}+k_{D_{a}D_{b}}+k_{D_{a}D_{c}}+\cdots \;\right)D_{a}\left(t\right)+k_{D_{b}D_{a}}D_{b}\left(t\right)\\ & & +\;k_{D_{c}D_{a}}D_{c}\left(t\right)+\cdots \\ \frac{dD_{b}\left(t\right)}{dt} & = & -\left(k_{D}+k_{D_{b}A}+k_{D_{b}D_{a}}+k_{D_{b}D_{c}}+\cdots \;\right)D_{b}\left(t\right)+k_{D_{a}D_{b}}D_{a}\left(t\right)\\ & & +\;k_{D_{c}D_{b}}D_{c}\left(t\right)+\cdots \\ & & \vdots \\ \frac{dA(t)}{dt} & & =-k_{A}+k_{D_{a}A}D_{a}\left(t\right)+k_{D_{b}A}D_{b}\left(t\right)+\cdots \\ & & \left(a,\;b,\;c,\;\cdots =1,\;2,\;3,\;\cdots \;n,\;a\;\neq \;b\;\neq \;c\;\neq \;\cdots \right) \end{array}$$

where D*_i_*(*t*) and A(*t*) denote the excited‐state populations of the *i*th donor and the acceptor, respectively; *k*_DA_ and *k*_DD_ denote the rate constants of FRET and homo‐FRET, respectively; *k*_D_ and *k*_A_ are the inverse of intrinsic lifetimes of the singlet excited states of the donor and the acceptor, respectively.

Prior to solving the multiple differential equations shown in Equation (2), the rate constants (e.g., *k*_D_, *k*_DaA_, *k*_DaDb_) included in the equations must be determined. Each rate constant is determined by *k*_D_, *κ* ^2^, and *Δ*_DA_ (and *Δ*_DD_), as shown in the following equation

(3) $$\begin{array}{c} \begin{array}{c} k_{D_{a}A}\;=\;\frac{3}{2}\;\times \;k_{D}\;\times \;\kappa^{2}\;\times \;\frac{1}{{\Delta_{\mathrm{DA}}}^{6}},\;\;\left(a\;=\;1,\;2,\;3,\;\cdots \;n\right)\\ k_{D_{a}D_{b}}\;=\;\frac{3}{2}\;\times \;k_{D}\;\times \;\kappa^{2}\;\times \;\frac{1}{{\Delta_{\mathrm{DD}}}^{6}},\;\;\left(a,\;b\;=\;1,\;2,\;3,\;\cdots \;n,\;a\;\neq \;b\right)\\ {\Delta_{\mathrm{DA}}}^{6}\;=\;{\left(\frac{d_{\mathrm{DA}}}{{\bar{R}}_{0}\left(\mathrm{DA}\right)}\right)}^{6},\;{\Delta_{\mathrm{DD}}}^{6}\;=\;{\left(\frac{d_{\mathrm{DD}}}{{\bar{R}}_{0}\left(\mathrm{DD}\right)}\right)}^{6}\\ \;{\bar{R}}_{0}{\left(\mathrm{DA}\right)}^{6}=\frac{3\ln10}{64\pi^{5}N_{A}}\;\frac{Q_{D}}{n^{4}}J_{\mathrm{DA}},\;\;\;{\bar{R}}_{0}{\left(\mathrm{DD}\right)}^{6}=\frac{3\ln10}{64\pi^{5}N_{A}}\;\frac{Q_{D}}{n^{4}}J_{\mathrm{DD}} \end{array} \end{array}$$

where *κ* ^2^ is the orientation factor, which is the relative orientation between the transition dipole moments of the donor and acceptor chromophores; *d*_DA_ and *d*_DD_ are the D–A and D–D distances, respectively; *Δ*_DA_ and *Δ*_DD_ are normalized interchromophore distances obtained by dividing *d*_DA_ and *d*_DD_ by the Förster radius for the D–A or D–D pair, respectively; *R̄*_0_ is Förster radius when *κ* ^2^ is ⅔; *n* is the refractive index of the solvent and was set to be a constant; *N*_A_ is Avogadro's number; *Q*_D_ is the quantum yield of the donor emission; *J* is the spectral overlap integral between the emission spectrum of the donor and the absorption spectrum of the acceptor. Since FRET is a mechanism for describing energy transfer in the regime of weak electronic coupling, the electronic interactions between all of the chromophores were assumed to be weak and therefore *Q*_D_ and *J* were set to be constants. In contrast, *d*_DA_, *d*_DD_, and *κ* ^2^ were adjusted as major variables in the simulations to examine the effect of *n*_D_ and *d*_DD_ (and *d*_DA_) on *η*_FRET_. *k*_D_ was set to be 1000 ps^−1^ in the simulations. The orientation factor (*κ* ^2^) is defined as follows

(4) $$\begin{array}{c} \kappa^{2}=\left(1+3\cos^{2}\theta \right)\cos^{2}\omega \end{array}$$

where the angle *θ* represents the angular position of the acceptor relative to the donor's transition dipole vector, and *ω* is the angle between the acceptor transition dipole vector and the electric field of the donor at the location of the acceptor. Since these angles are assumed to be randomly distributed at the isotropic limit, *κ* ^2^ for each FRET pathway can be represented by two random numbers. For cases (i) and (iii) corresponding to the dynamic isotropic limit, *κ* ^2^ value was set to be ⅔, which is the average value of the isotropic distribution. For cases (ii) and (iv) corresponding to the static isotropic limit (Figure S12, Supporting Information), random numbers were generated for the two angles (that is, *θ* and *ω*) within the range of 0–2*π*, and calculated *κ* ^2^ with Equation (4). The approach of calculating *η*_FRET_ at the dynamic and static isotropic limits used in the simulations is similar to the one used in previous studies on the FRET behavior at those limits.^[^ ^21^, ^23^ ^]^

##### Frequency‐Resolved Femtosecond Transient Absorption Spectroscopy

TA spectra were measured with femtosecond laser pulses using a visible pump–broadband probe scheme. The femtosecond pulses at the center wavelength of 800 nm were generated from a Ti:sapphire amplified laser (Coherent Legend Elite) and split into pump and probe beams. On the pump arm, the 800 nm pulses were converted into the 540 nm pulses with the bandwidth of 16 nm using a home‐built, all‐reflective‐optic noncollinear optical parametric amplifier. Since, at 540 nm wavelength, the absorption of the donor is at least 8 times larger than that of the acceptor, the pump wavelength was selected of 540 nm to preferentially excite donors. The pump pulses were sent through a pair of Brewster‐cut fused‐silica prisms to precompensate for the dispersion obtained from transmissive optics and compressed to near‐transform‐limited pulses at the sample position. The pulse energy of the pump pulses was varied from 50 to 200 nJ to check the dependence of transient absorption signal on the excitation energy. It should be noted that, to prevent the excitation of multiple donors, the pump pulses with the pulse energy at the minimal level (50 nJ per pulse) were used for the measurement of TA spectra. On the probe arm, the laser pulses at 800 nm were sent into a sapphire window of 3 mm thickness and converted into white light continuum. The visible portion (460–725 nm) of the white light continuum was used as broadband probe pulses without further compensation of the dispersion. The probe pulses were time‐delayed with respect to the pump pulses using a motorized translation stage (Newport, M‐ILS150HA). By recording the “pump‐on” and “pump‐off” probe spectra, the differential transmission (*Δ* *T*/*T*) spectra were obtained as a function of time. The spectra of transient signal and the reference were detected by a spectrometer (Andor, SR303i) equipped with a CCD (Andor, DU420A). In all the TA measurements, the polarization of the pump pulses was set to be at the magic angle (54.7°) relative to the probe polarization in order to prevent the contribution of anisotropic components of the TA signal. For the TA measurement, porphyrin dendrimers were dissolved in tetrahydrofuran (THF). The porphyrin dendrimer solution in THF was prepared with an optical density of ≈0.4 at its absorption maximum of 543 nm in THF in a glass cuvette of 1 mm thickness.

##### Statistical Analysis

Microsoft Excel and OriginPro were used for the statistical analysis of the data presented in this work. The preprocessing and data presentation were performed in the following way. Data in Figure 2c,d represent the averaged *η*_FRET_ of an individual donor, obtained from 100,000 simulations. The standard error of the mean (SEM) of the *η*_FRET_ is less than 0.05%. The mean ± SD of data in Figure 4a are 1.69 ± 0.45 nm. Figure 4b shows mean ± SD (1.69 ± 0.44 nm). The mean ± SD of data in Figure 4c are 1.92 ± 0.40 nm (top panel), 2.34 ± 0.61 nm (middle panel), and 2.85 ± 0.76 nm (bottom panel). Table 1 shows mean ± SD for time constants and *β* values, which were determined from the global fits of the first and second right singular vectors of TA spectra. Table 1 also shows mean ± SD of the experimental *η*_FRET_. The mean values of theoretical FRET efficiency and modified theoretical *η*_FRET_ are shown in Table 1, and their SEMs are less than 0.05%.

## Conflict of Interest

The authors declare no conflict of interest.

## Supporting information

Supporting Information
