# Supplementary material for: Enhancement of Energy Transfer Efficiency with Structural Control of Multichromophore Light‐Harvesting Assembly
Source: Adv Sci (Weinh). 2020 Aug 19;7(20):2001623. doi: 10.1002/advs.202001623 (PMC7578888; doi:10.1002/advs.202001623)
Supplement: Supplementary file 1 — Supporting Information [file ADVS-7-2001623-s001.pdf]

© 2020 Wiley-VCH GmbH

ADVANCED SCIENCE
Open Access

Supporting Information

for *Adv. Sci.,* DOI: 10.1002/advs.202001623

Enhancement of Energy Transfer Efficiency with Structural Control of Multichromophore Light-Harvesting Assembly

Inhwan Oh, Hosoowi Lee, Tae Wu Kim, Chang Woo Kim, Sunhong Jun, Changwon Kim, Eun Hyuk Choi, Young Min Rhee, Jeongho Kim, Woo-Dong Jang,* and Hyotcherl Ihee**

Supporting Information

Enhancement of Energy Transfer Efficiency with Structural Control of Multi-Chromophore Light-Harvesting Assembly

Inhwan Oh, Hosoowi Lee, Tae Wu Kim, Chang Woo Kim, Sunhong Jun, Changwon Kim, Eun Hyuk Choi, Young Min Rhee, Jeongho Kim, Woo-Dong Jang,* and Hyotcherl Ihee**

Table of contents

SI Note 1. Synthesis and characterization of multi-porphyrin dendrimers

SI Note 2. Steady-state absorption/emission spectra of multi-porphyrin dendrimers

SI Note 3. Molecular dynamics simulations for multi-porphyrin dendrimers.

SI Note 4. Experimental determination of FRET efficiencies in multi-porphyrin dendrimers

SI Note 5. Excitation power dependence of TA signals

SI Note 6. Calculation of theoretical FRET efficiencies in multi-porphyrin dendrimers

SI Note 7. Enhancement of FRET efficiency in the model MCSs examined with simulations

SI Note 8. Enhancement of FRET efficiency dependent on normalized D-A distance

SI Note 9. Comparison with previous studies

SI Note 10. Principle for prediction of FRET efficiency for multi-chromophore systems

SI Figures

SI References

SI Note 1. Synthesis and characterization of multi-porphyrin dendrimers

Materials and measurements

All commercially available chemicals were reagent grade purchased from Sigma-Aldrich, TCI, and Alfa Aesar and used without further purification. Air-/water-sensitive reagents were treated under nitrogen atmosphere with anhydrous condition. Preparative recycling size exclusion chromatography (SEC) was performed on JAI model LC9021 equipped with JAIGEL-1H, JAIGEL-2H and JAIGEL-3H columns using $CHCl_3$ (HPLC grade / J.T. Baker) as an eluent. ^{1}H and ^{13}C NMR spectroscopy was performed on a Bruker Avance DPX 400 spectrometer at ambient temperature. Matrix-assisted-laser-desorption-ionization time-of-flight mass (MALDI TOF MS) spectrometry was performed a Bruker Daltonics LRF20 mass spectrometer with dithranol (1,8,9-trihydroxyanthracene) as a matrix.

Synthesis and characterization

Some of compounds, such as **$(OMe)_6(OH)_2P_{FB}$**, **$(OMe)_8P_{FB}$**, **$(OH)_8P_{FB}$**, **P1**, **P2**, **P3**, **P5**, **SD4A**, and **SD16A**, were synthesized according to the previously reported methods.[1] *Cis*-**$(OMe)_4(OH)_4P_{FB}$** and *trans*-**$(OMe)_4(OH)_4P_{FB}$** were synthesized with the same synthetic procedure as **$(OMe)_6(OH)_2P_{FB}$**, except the reactant ratio between pyrrole, 3,5-dimethoxybenzaldehyde, and 3,5-dihydroxybenzaldehyde. Synthetic procedures for all compounds are illustrated in Schemes S1 and S2.

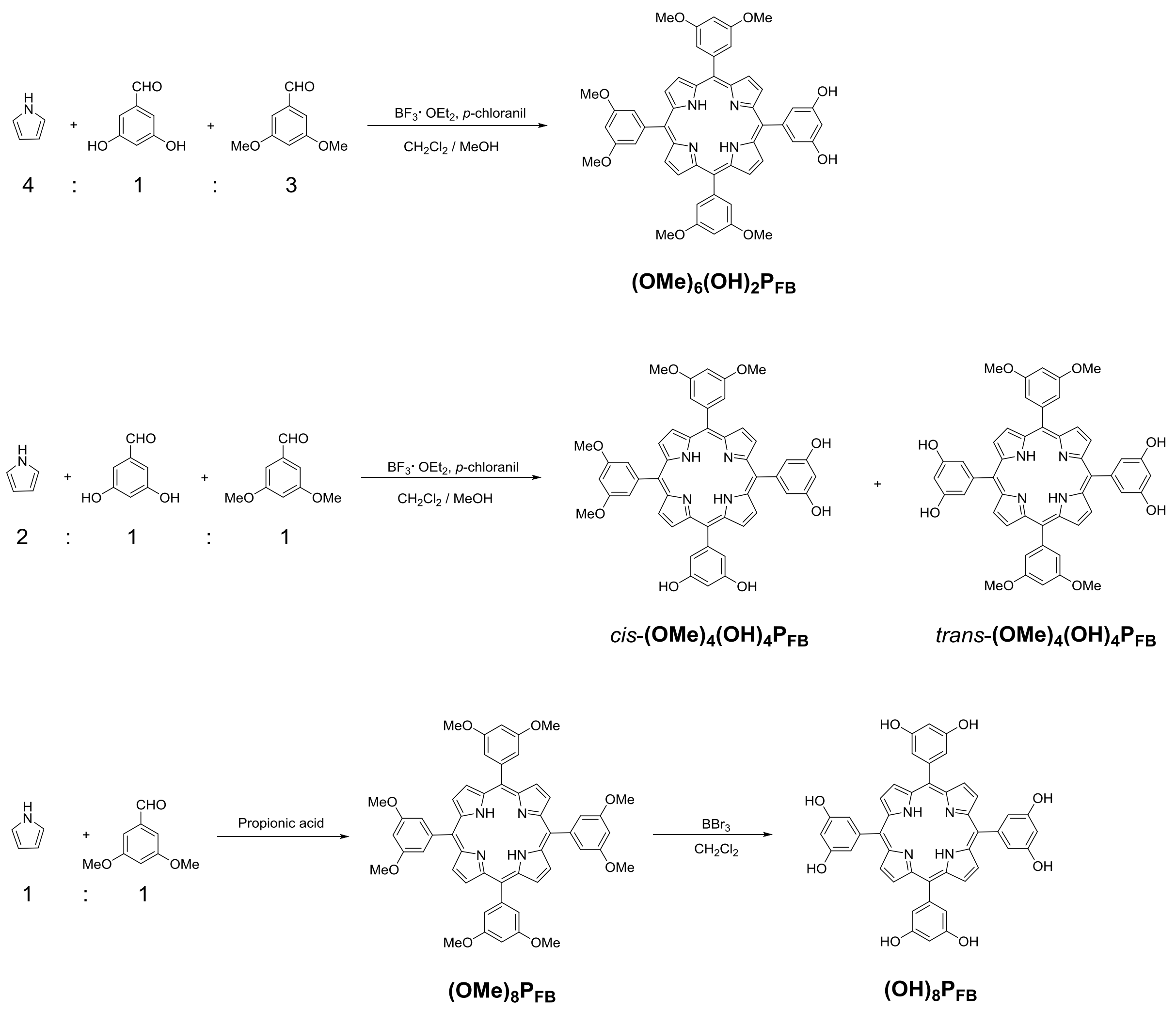

Scheme S1. Synthetic procedure for free-base porphyrins.

Cis-**$(OMe)_4(OH)_4P_{FB}$** and *trans*-**$(OMe)_4(OH)_4P_{FB}$**: To a mixture solution of 3,5-dihydroxybenzaldehyde (300 mg, 2.17 mmol) and 3,5-dimethoxybenzaldehyde (361 mg, 2.17 mmol) in $CHCl_3$/MeOH (10:1) mixture solvent (200 mL), pyrrole (300 μL, 4.34 mmol) and BF3·OEt2 (0.2 mL, 1.62 mmol) were added and stirred for 30 m at 0 °C and 12 h at 25 °C, respectively, under N_2 atmosphere. Then, *p*-chloranil (854 mg, 3.47 mmol) was added to the reaction mixture and further stirred for 12 h at 25 °C. The reaction mixture was evaporated to dryness and subjected to flash silica column chromatography using 5 % MeOH containing CH_2Cl_2 as the eluent to remove insoluble impurities. Further purification was conducted

through gradient column chromatography with gradually increasing ratio of MeOH (0 – 5 %) in CH_2Cl_2 as eluent. Every fraction was collected separately and subjected to MALDI TOF MS to confirm molecular weight of each fraction. From the result of MALDI TOF MS, the third and fourth fractions were matched with the molecular weight of **$(OMe)_4(OH)_4P_{FB}$**. The third and fourth fractions was evaporated to dryness and recrystallized from THF/n-hexane to give purple solids. Although both *trans*-**$(OMe)_4(OH)_4P_{FB}$** and *cis*-**$(OMe)_4(OH)_4P_{FB}$** showed similar ^{1}H NMR spectra, after the coupling reaction with **P4**, the third and fourth fractions were identified as *trans*-**$(OMe)_4(OH)_4P_{FB}$** and *cis*-**$(OMe)_4(OH)_4P_{FB}$**, respectively.

Cis-**$(OMe)_4(OH)_4P_{FB}$**: Isolation yield (50 mg, 6 %). ^{1}H-NMR (400 MHz, CD_3OD): δ = 8.96 (8 H; overlapped peak of pyrrolic-β *H* in P_{FB}), 7.37 (s, 4 H; *o*-C_6H_3), 7.15 (s, 4 H; *o*-C_6H_3), 6.95 (s, 2 H; *p*-C_6H_3), 6.72 (s, 2 H; *p*-C_6H_3). MALDI TOF MS for $C_{48}H_{38}N_4O_8$ m/z: caldc. 798.84, [M^+]; found.799.934.

Trans-**$(OMe)_4(OH)_4P_{FB}$**: Isolation yield (7.5 mg, 1 %). ^{1}H-NMR (400 MHz, CD_3OD): δ = 8.96 (8 H; overlapped peak of pyrrolic-β *H* in P_{FB}), 7.38 (s, 4 H; *o*-C_6H_3), 7.15 (s, 4 H; *o*-C_6H_3), 6.96 (s, 2 H; *p*-C_6H_3), 6.72 (s, 2 H; *p*-C_6H_3). MALDI TOF MS for $C_{48}H_{38}N_4O_8$ m/z: caldc. 798.84, [M^+]; found.799.908.

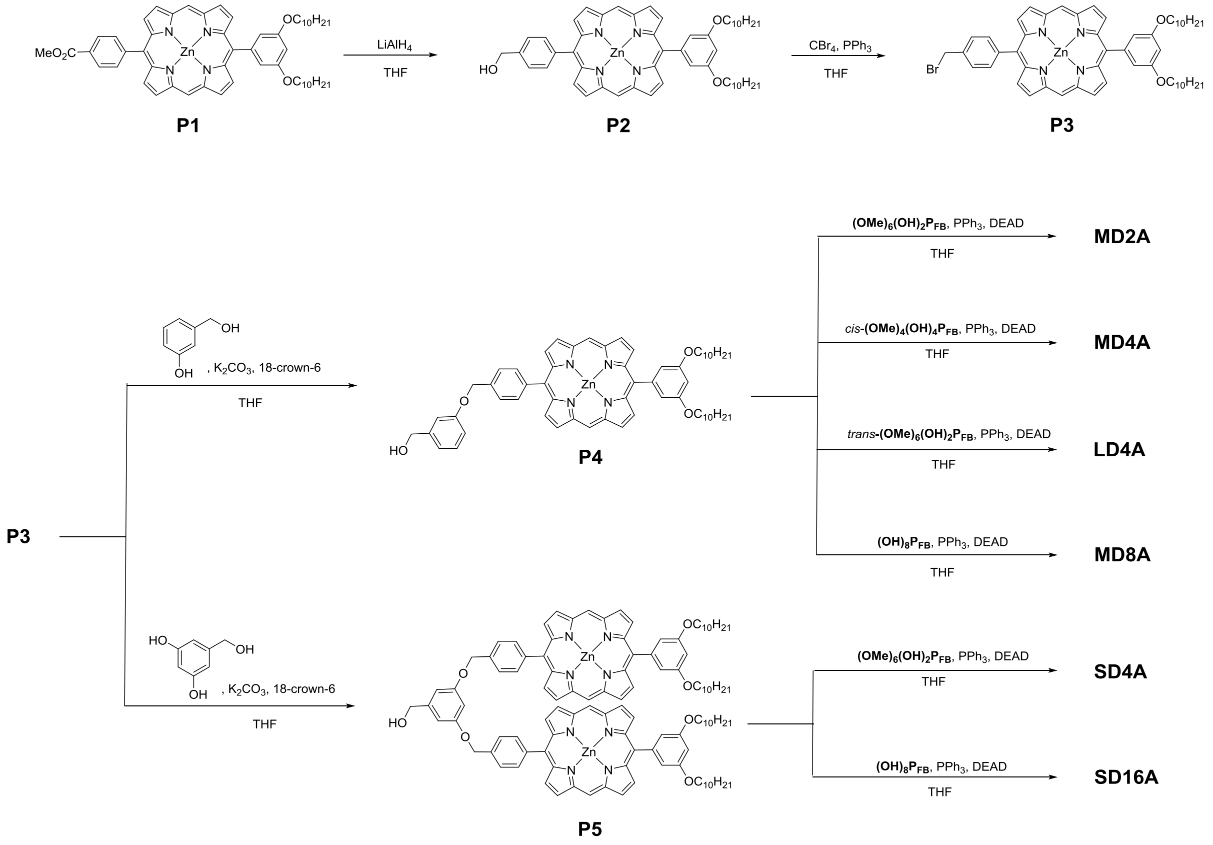

Scheme S2. Synthetic procedure for multi-porphyrin dendrimers.

P4: To a mixture solution of **P3** (688 mg, 0.792 mmol) and 3-hydroxybenzyl alcohol (76.0 mg, 0.612 mmol) in anhydrous THF (1.5 mL), K_2CO_3 (111 mg, 0.803 mmol) and 18-crown-6 ether (152 mg, 0.617 mmol) were added and refluxed for 2 days under N_2 atmosphere. The reaction mixture was evaporated to dryness. The residue was dissolved to CH_2Cl_2 and washed with distilled water. The combined organic layer was dried over Na_2SO_4. The crude solution was evaporated to dryness and purified by silica column chromatography with CH_2Cl_2 and ethyl acetate as the eluent to obtain **P4** as the form of bright red solid (615 mg, quantitative yield). ^{1}H-NMR (400 MHz, $CDCl_3$): δ = 10.33 (s, 2 H; meso-*H* in P_{Zn}), 9.45 (t, 2H; pyrrolic-β *H* in P_{Zn}), 9.28-9.27 (d, 2H; pyrrolic-β *H* in P_{Zn}), 9.18-9.16 (d, 2 H; pyrrolic-β *H* in P_{Zn}), 8.31-8.29 (d, 2 H; *o*-C_6H_4-P_{Zn}), 7.88-7.86 (d, 2 H; *m*-C_6H_4-P_{Zn}), 7.44-6.92 (m, 7 H; overlapped peaks of C_6H_4 and C_6H_3-P_{Zn}), 5.43 (s, 2 H; Ar-*CH₂*-O-Ar), 4.57-4.50 (d, 2 H; Ar-*CH₂*-OH),

4.17-4.13 (t, 4 H; -*OCH*$_2$-(CH$_2$)$_8$CH$_3$), 1.90-0.83 (m, 38 H; overlapped peaks of -*(CH$_2$)$_8$CH$_3$*). MALDI TOF MS for $C_{60}H_{68}N_4O_4Zn$ m/z: calcd. 974.59, [M^+]; found: 974.387.

MD2A: To a mixture solution of **(OMe)$_6$(OH)$_2$P$_{FB}$** (30.0 mg, 0.0363 mmol), **P4** (74.0 mg, 0.0759 mmol) and PPh_3 (20.0 mg, 0.0763 mmol) in dry THF (3.0 mL), 40 % solution of diethylazodicarboxylic acid (DEAD) in toluene (35 μL, 0.077 mmol) was slowly added and vigorously stirred at 0 °C for 30 m and 25 °C for 18 h under N_2 atmosphere. The reaction mixture was evaporated for dryness. The residue was dissolved in CH_2Cl_2 and washed with distilled water. The combined organic layer was dried over Na_2SO_4. The crude solution was evaporated to dryness and purified by silica column chromatography with CH_2Cl_2 as the eluent. The first fraction was collected and further purified with preparative recycle SEC. The bright red powder of **MD2A** was obtained by freeze-drying from benzene in 17 % yield (16.8 mg). ^{1}H-NMR (400 MHz, $CDCl_3$): δ = 9.99 (s, 4 H; meso-*H* in P_{Zn}), 9.26-8.92 (m, 24 H; overlapped peaks of pyrrolic-β *H* in P_{Zn} and P_{FB}), 7.69-7.62 (d, 4 H; *o*-C_6H_4-P_{Zn}), 7.44-7.43 (d, 4 H; *m*-C_6H_4-P_{Zn}), 7.33-6.72 (m, 26 H; overlapped peaks of C_6H_4, C_6H_3-P_{Zn}, and C_6H_3-P_{FB}), 5.26 (s, 4 H; P_{Zn}-Ar-*CH$_2$*-O-Ar), 5.19 (s, 4 H; Ar-*CH$_2$*-O-Ar-P_{FB}), 4.17-4.13 (t, 8 H; -*OCH$_2$*-(CH$_2$)$_8$CH$_3$), 3.85 (s, 6 H; Ar-O-*CH3*), 3.78 (s, 12 H; Ar-O-*CH3*), 1.92-0.85 (m, 76 H; overlapped peaks of -*(CH$_2$)$_8$CH$_3$*), -2.72 (s, 2 H; -*NH* in P_{FB}). ^{13}C-NMR (100 MHz, $CDCl_3$): δ = 159.20, 158.87, 158.47, 158.18, 150.01, 149.91, 149.50, 144.30, 142.47, 138.61, 136.18, 132.76, 132.36, 129.92, 120.27, 119.52, 15.10, 114.73, 114.65, 10.21, 100.94, 100.27, 100.22, 70.43, 68.66, 66.89, 55.84, 32.09, 29.77, 29.67, 29.62, 29.53, 29.33, 22.88, 14.27. MALDI TOF MS for $C_{170}H_{174}N_{12}O_{14}Zn_2$ m/z: calcd. 2740.03, [M^+]; found: 2741.178.

MD4A: To a mixture solution of *cis*-**(OMe)$_4$(OH)$_4$P$_{FB}$** (30 mg, 0.038 mmol), **P4** (154 mg, 0.158 mmol), PPh_3 (42 mg, 0.158 mmol) in dry THF (3.0 mL), 40 % solution of DEAD in toluene (72 μL, 0.158 mmol) was slowly added and vigorously stirred for 1 h at 0 °C and 12 h at 25 °C under N_2 atmosphere. The reaction mixture was treated in a similar manner to that for **MD2A** to obtain **MD4A** as bright red powder in 2 % isolation yield (38 mg). ^{1}H-NMR (400 MHz, $CDCl_3$): δ = 9.76 (s, 8 H; meso-*H* in P_{Zn}), 9.14-8.79 (m, 40 H; overlapped peaks of pyrrolic-β *H* in P_{Zn} and P_{FB}), 7.93-6.87 (m, 56 H; overlapped peaks of all *aromatic-H*), 4.98-4.90 (d, 16 H; overlapped peaks of P_{Zn}-Ar-*CH$_2$*-O-Ar and Ar-*CH$_2$*-O-Ar-P_{FB}), 4.12-4.08 (t, 16 H; -*OCH$_2$*-$(CH_2)_8CH_3$), 3.69 (s, 12 H; -*OCH$_3$*), 1.86-0.81 (m, 76 H; overlapped peaks of -*(CH$_2$)$_8$CH$_3$*), -2.59 (s, 2 H; -*NH* in P_{FB}). ^{13}C-NMR (100 MHz, $CDCl_3$): δ = 158.72, 158.63, 458.28, 157.89, 149.72, 149.52, 149.16, 148.94, 135.79, 134.63, 132.40, 132.02, 131.48, 131.33, 125.58, 119.74., 119.17, 114.56, 113.89, 113.78, 105.80, 105.76, 77.23, 669.99, 68.40, 55.45, 31.88, 29.59, 29.56, 29.46, 29.42, 29.31, 26.13, 22.66, 14.10. MALDI-TOF MS for $C_{288}H_{302}N_{20}O_{20}Zn_4$ m/z: calcd. 4625.12, [M$^+$]; found: 4628.673.

LD4A: To a mixture solution of *trans*-**(OMe)$_4$(OH)$_4$P$_{FB}$** (7.4 mg, 0.092 mmol), **P4** (38 mg, 0.039 mmol), PPh_3 (10 mg, 0.62 mmol) in dry THF (1.5 mL), 40 % solution of DEAD in toluene (18 μL, 0.039 mmol) was slowly added and vigorously stirred for 0.5 h at 0 °C and 12 h at 25 °C under N_2 atmosphere. The reaction mixture was treated in a similar manner to that for **MD2A** to obtain **LD4A** as bright red powder in 2 % isolation yield (10 mg). ^{1}H-NMR (400 MHz, $CDCl_3$): δ = 9.91 (s, 8 H; meso-*H* in P_{Zn}), 9.21-8.84 (m, 40 H; overlapped peaks of pyrrolic-β *H* in P_{Zn} and P_{FB}), 7.92-6.88 (m, 56 H; overlapped peaks of all *aromatic-H*), 5.05-4.88 (d, 16 H; overlapped peaks of P_{Zn}-Ar-*CH$_2$*-O-Ar and Ar-*CH$_2$*-O-Ar-P_{FB}), 4.13-4.09 (t, 16 H; -*OCH$_2$*-$(CH_2)_8CH_3$), 3.63 (s, 12 H; -*OCH$_3$*), 1.90-0.81 (m, 76 H; overlapped peaks of -

(CH$_2$)$_8$CH$_3$), -2.66 (s, 2 H; -*NH* in P$_{FB}$). ^{13}C-NMR (100 MHz, CDCl$_3$): δ = 158.49, 149.99, 149.82, 149.48, 149.32, 134.90, 132.73, 132.30, 131.76, 131.667, 127.74, 125.85, 114.72, 114.09, 106.07, 101.02, 77.44, 70.28, 68.67, 68.59, 67.87, 55.59, 32.08, 29.92, 29.88, 29.79, 29.76, 29.67, 29.62, 29.52, 26.34, 14.31. MALDI-TOF MS for $C_{288}H_{302}N_{20}O_{20}Zn_4$ m/z: calcd. 4625.12, [M$^+$]; found: 4627.461.

MD8A: To a mixture solution of **(OH)$_8$P$_{FB}$** (51.0 mg, 0.0687 mmol), **P4** (604 mg, 0.620 mmol), PPh$_3$ (163 mg, 0.621 mmol) in dry THF (1.0 mL), 40 % solution of DEAD in toluene (280 μL, 0.616 mmol) was slowly added and vigorously stirred for 1 h at 0 °C and 12 h at 25 °C for under N$_2$ atmosphere. The reaction was monitored by thin layer chromatography and additional PPh$_3$ (163 mg, 0.621 mmol) and 40 % solution of DEAD in toluene (280 μL, 0.616 mmol) were added to the reaction mixture at 0 °C and further stirred at 25 °C for 2 h. The reaction mixture was treated in a similar manner to that for **MD2A** to obtain **MD8A** as bright red powder in 17 % yield (100 mg). ^{1}H-NMR (400 MHz, CDCl$_3$): δ = 9.53-8.58 (m, 88 H; overlapped peaks of meso-*H* in P$_{Zn}$ and pyrrolic-β *H* in P$_{Zn}$ and P$_{FB}$), 7.66-6.38 (m, 100 H; overlapped peaks of all *aromatic-H*), 4.55-4.45 (d, 32 H; overlapped peaks of P$_{Zn}$-Ar-*CH$_2$*-O-Ar and Ar-*CH$_2$*-O-Ar-P$_{FB}$), 4.05 (t, 32 H; -*OCH$_2$*-(CH$_2$)$_8$CH$_3$), 1.81-0.89 (m, 304 H; overlapped peaks of -*(CH$_2$)$_8$CH$_3$*), -2.25 (s, 2 H; -*NH* in P$_{FB}$). ^{13}C-NMR (100 MHz, CDCl$_3$): δ = 158.35, 157.59, 149.69, 149.34, 149.05, 018.49, 144.49, 135.51, 134.66, 132.4, 131.97, 131.53, 131.28, 125.662, 119.72, 119.05, 119.48, 113.47, 105.68, 100.87, 69.81, 68.82, 68.52, 8.23, 32.07, 29.75, 29.64, 29.54, 29.51, 26.28, 22.86, 14.33, 14.00. MALDI-TOF MS for $C_{524}H_{558}N_{36}O_{32}Zn_8$ m/z: calcd. 8395.30, [M$^+$]; found: 8403.855.

SI Note 2. Steady-state absorption/emission spectra of multi-porphyrin dendrimers

The steady-state absorption and emission spectra of the multi-porphyrin dendrimers and the monomeric units of donor and acceptor were measured using a UV/VIS spectrometer Shimadzu UV-2600 and a FL spectrometer Horiba QM-400, respectively, as shown in Figure S8. Figures S8c - S8h show that the absorption spectra of the multi-porphyrin dendrimers are well fit with a linear combination of the absorption spectra of the monomeric units of donor and acceptor. The donor-to-acceptor ratio determined from a linear combination fit of the absorption spectrum of each dendrimer is equal to the donor-to-acceptor number ratio predicted based on the chemical structure of the dendrimer. These results confirm that the electronic couplings between porphyrins are weak and the spectral overlap integral does not change despite the variation of n_D and d_{DD}. The energy transfer between porphyrins would occur only through the FRET mechanism because of weak electronic coupling between porphyrins.

SI Note 3. Molecular dynamics simulations for multi-porphyrin dendrimers.

The bonded parameters of the MD force field were generated by using the generalized AMBER force field (GAFF),[2] while the non-standard zinc atom of the donor molecule was connected to the nitrogen atoms by a harmonic bond potential of r_0 = 0.2042 nm and k = 3.8585×10^5 kJ mol^{-1} nm^{-1}. The electrostatics were modelled by disassembling the dendrimer molecules into fragments, and fitting the atomic partial charges of each fragment by the restrained electrostatic potential (RESP) method.[3] The structures of the fragment molecules are displayed in Figure S9. The electrostatic potential around the fragments were evaluated by Q-Chem 4.0 quantum chemistry package,[4] with DFT/B3LYP/6-31G(d,p) level of theory. Then the electrostatic potentials were converted to the atomic partial charges. The dispersion parameters were taken from GAFF, with the zinc parameters from AMBER99 force field.

For all MD simulations, one dendrimer molecule was placed in a cubic box of 7.0 nm side length and was solvated by tetrahydrofuran (THF) molecules. The THF molecules were parametrized by using GAFF and RESP, in a similar way to the dendrimer molecules. In the room temperature, the conformational change of the dendrimer appeared to be too slow, especially for molecules with large number of branches (MD8A and SD16A). Therefore, we facilitated the conformational sampling by a two-step procedure. First, a single trajectory was run at an elevated temperature, at which crossing the free energy barriers between local minima is much easier than in room temperature. After running the simulation for a sufficiently long time, the snapshots along the trajectory were gathered and separately used as initial conditions for relatively short room temperature simulations. The actual conformation pools for the analysis were formed from these room temperature trajectories.

The high-temperature simulations were run at 800 K in the canonical (constant NVT) condition for 105 ns for SD4A and MD4A, and 55 ns for other dendrimers. After truncating the first 5 ns of the trajectory as the equilibration period, snapshots of the system were collected every 100 ps. Each snapshot was cooled down during 100 ps in isothermal-isobaric (constant NPT) condition with 293 K and 1 bar, and then subjected to another 500 ps of production run in the same condition. During the production run, snapshots of the trajectory were saved every 1 ps. For all simulations, the temperature and the pressure were maintained by velocity-rescale thermostat[5] and Parrinello-Rahman barostat,[6] respectively. The electrostatic and dispersion interactions were treated via force shifting with 12 Å cutoffs with the buffering region of 2 Å, together with periodic boundary conditions. The time step for numerical integration was 1 fs at 800 K, and 2 fs at 293 K.

We defined d_{DA} as the distance between the center of the free-base porphyrin (acceptor) and zinc atom in each zinc porphyrin (donor), and d_{DD} as the distance from a zinc

atom to the zinc atom in each zinc porphyrin. The d_{DA} and d_{DD} distributions and mean values obtained from structures calculated by MD simulations are shown in Figure 4. Structures with d_{DA} or d_{DD} less than 0.5 nm were excluded because they are incompatible with the absorbance spectra. Similarity of d_{DA} distributions for all donors shows that each donor can be freely located within a certain distance range, that is, within the linker allowed range. The peak of d_{DA} distribution is not located in the middle of the distance distribution but has an asymmetric shape weighted toward longer distances, which seems to be due to the spherical shape of the dendrimer. This is because the farther d_{DA} is, the more space the donor can be located in. In summary, d_{DA} distribution obtained from the MD simulations shows that the dendrimer exists in a spherical form and the mean d_{DA} is approximately 1.69 ± 0.44 nm for all dendrimers.

SI Note 4. Experimental determination of FRET efficiencies in multi-porphyrin dendrimers

In general, η_{FRET} is determined by measuring (i) the change in the fluorescence intensity of the donor or (ii) the change of the excited-state lifetime of the donor when the acceptor is absent or present. However, the former method is likely to overestimate η_{FRET} because the fluorescence intensity of the donor can be reduced by other photophysical processes besides FRET. Also, the former method requires that the fluorescence intensities of the donor should be compared with both the FRET sample (that is, MCSs containing donors and an acceptor) and the control sample (that is, monomers of donor) having the exactly same donor concentration. However, it is very difficult to accurately adjust the concentration of the donor because the absorption features of the zinc porphyrin donor and the free-base porphyrin acceptor significantly overlap with each other at visible wavelengths. In contrast, the excited-state lifetime of the donor does not change with the concentration of a sample solution. Thus,

we determined η_{FRET} by measuring the excited-state lifetime of the donor. For all of the excited-state lifetime measurements, the excitation light at 543 nm was used to preferentially excite the zinc porphyrin donor. From the measured excited-state lifetimes of FRET sample and donor-only sample, η_{FRET} can be determined as follows:

$$\eta_{\text{FRET}} = 1 - \tau_{\text{DA}}/\tau_{\text{D}} \tag{S1}$$

where τ_{DA} is the excited-state lifetime of the FRET sample and τ_{D} is the excited-state lifetime of the donor-only sample.

SI Note 5. Excitation power dependence of TA signals

Exciton-exciton annihilation (EEA) is a common phenomenon observed in multi-chromophore systems, including zinc porphyrin dendrimers.[7,8] To check whether the TA signals of the multi-porphyrin dendrimers contain any contribution of EEA, we measured the TA signals of SD4A, which have the smallest d_{DD} among the dendrimers investigated in our study, while varying the pulse energy of the pump pulse, as shown in Figure S11. As a control experiment, we also measured the TA signal of SD4, a multi-porphyrin dendrimer consisting of only four donors without any acceptor, so that the TA signal without any contribution of FRET can be measured. The results show that the TA signals of both SD4A and SD4 decay more rapidly as the pulse energy of the pump pulse increases, indicating that the EEA occurs with the excitation of high fluence. To minimize the contribution of EEA, the TA spectra were measured with the pump pulses of 50 nJ energy.

SI Note 6. Calculation of theoretical FRET efficiencies in multi-porphyrin dendrimers

To calculate the theoretical η_{FRET} of the multi-porphyrin dendrimers using the simulations, we first need to determine which case, among (i) to (iv), the multi-porphyrin dendrimers belong

to. Since the linkers connecting the donors and the acceptor are composed of many single bonds, each donor will be in an arbitrary orientation with respect to the acceptor. However, it would be difficult for the donors to rotate rapidly because of long hydrocarbon chains. According to the MD simulations on the multi-porphyrin dendrimers, d_{DA} and d_{DD} are distributed as shown in Figure 4. Thus, the multi-porphyrin dendrimers investigated in this study correspond to case (iv). To calculate the theoretical η_{FRET}'s of multi-porphyrin dendrimers, it is necessary to know the shapes of distributions and the mean values of the normalized inter-chromophore distances (that is, Δ_{DA} and Δ_{DD}) for all the chromophore pairs composing the dendrimers. Δ_{DA} can be calculated by dividing the mean d_{DA} obtained from the MD simulations by the Förster radius of the D-A pair. To calculate the Förster radius of the D-A pair, the emission quantum yield of the donor and the spectral overlap integral between the donor and the acceptor in THF solvent were determined to be 3.3 % and 3.93×10^{14} nm^4 M^{-1} cm^{-1}, respectively, from static emission measurement. These values are similar to those reported in previous studies on zinc porphyrin.[9,10] Based on these values and the refractive index of THF (1.404), the Förster radius of the D-A pair and Δ_{DA} were calculated to be 2.43 nm and 0.7, respectively, using Equation (3) in the Methods section. In the same manner, the Förster radius of the D-D pair and Δ_{DD} were calculated. The Förster radius for the D-D pair was determined to be 1.92 nm, and Δ_{DD} was determined to be 1.0 for the short D-D pair, 1.2 for the medium D-D pair, and 1.5 for the long D-D pair. Finally, the theoretical η_{FRET} of the multi-porphyrin dendrimers were calculated from the simulations considering the distributions and the mean values of Δ_{DA} and Δ_{DD}, as shown in Table 1. Since there are too many D-A and D-D pairs in SD16A, it was difficult to consider them all. Therefore, we estimated that the

theoretical η_{FRET} of SD16A lies in the range between η_{FRET}'s of D12A and D20A when Δ_{DD} is 1.0.

SI Note 7. Enhancement of FRET efficiency in the model MCSs examined with simulations

While Δ_{DA} is the main factor that determines η_{FRET}, the distribution of Φ_{FRET} reflects structural heterogeneity in an MCS and thus is a good measure of estimating the degree of enhancement of η_{FRET} (that is, $\Delta\eta_{FRET}$) aided by homo-FRET. When multiple donors in an MCS have different Φ_{FRET} values from each other, D-D energy transfer (that is, homo-FRET) would activate the detour FRET paths of high efficiencies. Therefore, as the distribution of Φ_{FRET} becomes broader, homo-FRET would enhance η_{FRET} further, resulting in a larger $\Delta\eta_{FRET}$ value.

Here we need to consider the origins of the distribution of Φ_{FRET}. The distribution of Φ_{FRET} arises from different origins in cases (ii) - (iv) investigated in the present study. In case (ii), the distribution of Φ_{FRET} is governed by the distribution of κ^2. As can be seen in Figure S12, the distribution of κ^2 is asymmetric with a large population around $\kappa^2 = 0$. As a result of such asymmetric distribution of κ^2, the distribution of Φ_{FRET} is also asymmetric and largely populated below its mean value, η_{FRET}. Therefore, at large Δ_{DA} values where η_{FRET} is small, the distribution of Φ_{FRET} tends to be narrow and, at small Δ_{DA} values where η_{FRET} is large, the distribution of Φ_{FRET} tends to be broad. For example, the distributions of Φ_{FRET} at various Δ_{DA} values in case (ii) are shown in Figure S15. It can be seen that the distribution of Φ_{FRET} is the narrowest at $\Delta_{DA} = 1.6$ and the broadest at $\Delta_{DA} = 0.8$. Due to the narrow distribution of Φ_{FRET}

at $\Delta_{DA} > 1.5$, $\Delta\eta_{FRET}$ is negligibly small in that Δ_{DA} region, as can be seen in Figure S14j. To examine how the distribution of Φ_{FRET} influences $\Delta\eta_{FRET}$, we compared $\Delta\eta_{FRET}$ and the width of Φ_{FRET} distribution with respect to Δ_{DA}. The width of Φ_{FRET} distribution can be quantified by the variance of the distribution. As can be seen in Figure S17a, $\Delta\eta_{FRET}$ and the variance of Φ_{FRET} distribution are in good agreement with each other, indicating that the $\Delta\eta_{FRET}$ value at a given Δ_{DA} is governed by the width of Φ_{FRET} distribution.

In case (iii), the distribution of Φ_{FRET} is governed by the distribution of Δ_{DA}. Specifically, as the distribution of Δ_{DA} becomes broader, the distribution of Φ_{FRET} becomes broader and therefore $\Delta\eta_{FRET}$ increases, as shown in Figure S16. In our simulations, for all of the model MCSs, we set the width of the Δ_{DA} distribution to be constant with the relative standard deviation (RSD) of 20 %, irrespective of the mean Δ_{DA} value. As stated above, we remind that η_{FRET} is mainly governed by Δ_{DA}. As a result, as shown in Figure S14j - S14l, even with the constant width of the Δ_{DA} distribution, $\Delta\eta_{FRET}$ also varies depending on the value of Δ_{DA}. For example, in case (iii), when Δ_{DA} is varied by 0.1, FRET efficiency changes by only 0.1 % around $\Delta_{DA} = 0.2$ but changes by 12 % around $\Delta_{DA} = 1.0$, as can be seen in Figure S14k. This observation indicates that the distribution of Φ_{FRET} varies depending on the Δ_{DA} value, even when the width of Δ_{DA} distribution is constant. As was done for case (ii), we also compared $\Delta\eta_{FRET}$ and the width of Φ_{FRET} distribution with respect to Δ_{DA}, with the width of Φ_{FRET} distribution quantified by its variance. As shown in Figure S17b, $\Delta\eta_{FRET}$ and the variance of Φ_{FRET} distribution are in good agreement with each other. Therefore, at $\Delta_{DA} < 0.4$ where the distribution of Φ_{FRET} is narrow, $\Delta\eta_{FRET}$ is negligibly small, as can be seen in Figure

S14k. As mentioned in main text, in case (iv), the distribution of Φ_{FRET} is governed by distributions of both κ^2 and Δ_{DA}, as confirmed by the comparison of $\Delta\eta_{\mathrm{FRET}}$ for case (iv) with the sum of $\Delta\eta_{\mathrm{FRET}}$'s for cases (ii) and (iii) shown in Figure S18. Therefore, in case (iv), η_{FRET} is enhanced in the entire Δ_{DA} region.

SI Note 8. Enhancement of FRET efficiency dependent on normalized D-A distance

We examined how $\Delta\eta_{\mathrm{FRET}}$ changes depending on Δ_{DA}. When Δ_{DA} has a distribution of values as in case (iii), we found that the shape of $\Delta\eta_{\mathrm{FRET}}$ depending on Δ_{DA} is similar to the inverse of the 1st derivative of η_{FRET} with respect to Δ_{DA}, $-\mathrm{d}\eta_{\mathrm{FRET}} / \mathrm{d}\Delta_{\mathrm{DA}}$, as can be seen in Figure S14g and S14k. To confirm this idea, we compared the Δ_{DA} dependences of $\Delta\eta_{\mathrm{FRET}}$ and $-\mathrm{d}\eta_{\mathrm{FRET}} / \mathrm{d}\Delta_{\mathrm{DA}}$ in Figure S19c. As expected, it can be seen that $\Delta\eta_{\mathrm{FRET}}$ depending on Δ_{DA} is in good agreement with $-\mathrm{d}\eta_{\mathrm{FRET}} / \mathrm{d}\Delta_{\mathrm{DA}}$. In other words, when there exists structural heterogeneity of Δ_{DA}, $\Delta\eta_{\mathrm{FRET}}$ is determined by the 1st derivative of the Förster's equation with respect to Δ_{DA}. As a result, $\Delta\eta_{\mathrm{FRET}}$ is large at intermediate Δ_{DA} values where the slope of η_{FRET} with respect to Δ_{DA} is large, and $\Delta\eta_{\mathrm{FRET}}$ is small at small or large Δ_{DA} values where the slope of η_{FRET} with respect to Δ_{DA} is small. We also note that the curves of $\Delta\eta_{\mathrm{FRET}}$ depending on Δ_{DA} for all the model MCSs have the same shapes as each other, as can be seen in Figure S19b. Therefore, n_{D} only determines the overall degree of $\Delta\eta_{\mathrm{FRET}}$, not the shape of $\Delta\eta_{\mathrm{FRET}}$ depending on Δ_{DA}. This finding provides the underlying principle for the determination of $\Delta\eta_{\mathrm{FRET}}$ in the presence of structural heterogeneity of Δ_{DA}, that is, (i) the enhancement of η_{FRET}

by homo-FRET is governed by the 1st derivative of the Förster's equation with respect to Δ_{DA}, independent of n_D, and (ii) the magnitude of $\Delta\eta_{FRET}$ is determined by n_D.

We also examined how $\Delta\eta_{FRET}$ is influenced by the changes of n_D and Δ_{DD}. In case (ii) with a distribution of κ^2, homo-FRET among a large number of donors with various transition dipole orientations effectively serves as rapid rotational diffusion of chromophores, allowing the energy transfer to occur at the donors having favorable dipole orientations. Similarly, in case (iii) with a distribution of Δ_{DA}, homo-FRET among a large number of donors effectively serves as rapid translational diffusion of the donors, allowing the energy transfer to occur at donors having favorable Δ_{DA}'s. Actually, the enhancement of η_{FRET} due to the translational diffusion of donor and acceptor molecules has been already reported in previous studies.[11,12] Thus, the increase of n_D and the decrease Δ_{DD} can effectively increase the rate of homo-FRET, resulting in the increase of $\Delta\eta_{FRET}$. However, as the rate of homo-FRET reaches a certain limit, the enhancement of η_{FRET} becomes saturated, probably because the decrease of Δ_{DD} increases the number of detour paths no longer. Also, the enhancement of η_{FRET} with the increase of n_D becomes saturated. For example, as shown in Figure S20, as n_D increases, η_{FRET} curve of DnA in case (ii) converges the η_{FRET} curve of D1A in case (i), which corresponds to the dynamic isotropic limit. Thus, the increase of $\Delta\eta_{FRET}$ with the increase of n_D and the decrease Δ_{DD} is saturated at a certain limit.

SI Note 9. Comparison with previous studies

Our simulations can explain the seemingly conflicting results of the previous studies and well describes the FRET efficiency in MCSs. In agreement with our simulations, the $\Delta\eta_{FRET}$ with

the aid of homo-FRET has been already suggested in a previous study. According to a previous spectroscopic study on DNA-fluorophore systems,[13] it was experimentally demonstrated that the molecular system consisting of two donors and a single acceptor exhibits a higher η_{FRET} than the system consisting of a single donor and a single acceptor. Also, according to simulations performed on the same molecular systems, such increase of η_{FRET} in the molecular system with an additional donor is attributed to the occurrence of homo-FRET. Notably, the simulations in that previous study showed that η_{FRET} changes only by 3 % when d_{DD} is changed from 13 Å (Δ_{DD} = 0.25) to 20 Å (Δ_{DD} = 0.38), that is, 54 % increase, implying that η_{FRET} is not much affected by the rate of homo-FRET. Such insensitivity of η_{FRET} to Δ_{DD} can be explained by the results of our simulations. According to our simulations, the increase of $\Delta\eta_{\mathrm{FRET}}$ with the decrease of Δ_{DD} is saturated at $\Delta_{\mathrm{DD}} < 0.4$, as can be seen in Figure S14. This observation implies that the decrease of Δ_{DD} below a certain threshold does not increase the number of detour FRET paths via homo-FRET any further, as discussed in SI Note 8. In other words, any additional decrease of Δ_{DD} from the saturation limit does not significantly enhance η_{FRET}. Thus, the limited increase of $\Delta\eta_{\mathrm{FRET}}$ with the decrease of d_{DD} observed in this previous study suggests that Δ_{DD} was varied only in a narrow range.

Olejko and coworkers[14] showed the influence of n_{D} on η_{FRET} using MCSs consisting of multiple donors (FAM) and a single acceptor (Cy5) attached to the DNA origami structure, as mentioned in the Introduction section. They determined η_{FRET} from the measured fluorescence lifetime of the donor, while varying n_{D} from one to four, and concluded that the increase of n_{D} barely affects η_{FRET}. This observation can be easily rationalized by noting that

their MCSs correspond to conditions where η_{FRET} is hardly affected by n_{D}. In their molecular systems, since the dyes are rigidly fixed to the DNA origami structure at desired positions, the distances between the dyes would vary negligibly and the dipole orientations of the dyes are expected to change on much slower time scale than FRET. On the other hand, since it is difficult to achieve the perfect alignment of dipoles of dyes, the dyes are expected to have random orientations. Therefore, their MCSs correspond to case (ii). Based on the fact that the measured η_{FRET} was ~20 %, Δ_{DA} of these MCSs is estimated to be about 1.2. Our simulation results (see Figure S14j) show that the $\Delta\eta_{\mathrm{FRET}}$ is only ~2 % under these conditions since the FRET pathways from all donors are inefficient regardless of κ^2. We note that the simulation results shown in Figure S14j are for Δ_{DD} = 0.1. Since Δ_{DD} of the MCSs used in the previous study is estimated to be much longer than 0.1, η_{FRET} will be hardly enhanced with the increase of n_{D}. In contrast to the one-step FRET systems (FAM-Cy5), the two-step FRET systems (FAM-Cy3-Cy5), whose Δ_{DA} is reduced from 1.2 to 0.8 due to the increased spectral overlap, show ~10 % enhancement of η_{FRET} as the number of FAM-Cy3 increases. Thus, the MCSs investigated in that previous study are under the conditions where η_{FRET} is hardly affected by n_{D}.

Buckhout-White and coworkers[15] studied the effects of multiple donors on η_{FRET} using DNA networks including linear, bifurcated, Holliday junctions, and 8-arm star structures corresponding to case (ii) for the same reasons as the MCSs based on DNA origami study.[14] In that study, for 20 molecular systems whose Δ_{DA} was systematically varied, η_{FRET} was determined from the measurements of fluorescence from donors. For those systems, it was found that η_{FRET} does not show clear dependence on n_{D} even in MCSs with Δ_{DA} = 0.75 where the $\Delta\eta_{\mathrm{FRET}}$ is expected to be largest in case (ii). This result might be due to the low

formation efficiency of some of the MCSs used in that study. For example, the formation efficiencies of linear and bifurcated structures are very high (> 90 %) but those of 8-arm star structures and Holliday junction structure with Δ_{DA} = 0.75 are very low (< 20 %). In fact, when only the MCSs of high formation efficiencies are considered, it can be clearly seen that η_{FRET} is enhanced with the increase of n_D. In addition, for the MCSs of high formation efficiencies, Δ_{DA} dependence of $\Delta\eta_{FRET}$ is also in good agreement with the results of our simulations.

SI Note 10. Principle for prediction of FRET efficiency in multi-chromophore systems

The simulations presented in this work can be utilized to calculate the theoretical η_{FRET} of various MCSs. First, it is needed to determine which case, among cases (i) - (iv), an MCS of interest belongs to, based on the chemical structure of the MCS. The next step is to estimate the structural parameters of the MCS, for example mean values and distributions of normalized inter-chromophore distances (that is, Δ_{DA} and Δ_{DD}) between all chromophore pairs. The Förster radii of the D-A and D-D pair can be calculated by using steady-state spectra of the donor and the acceptor. The distributions of Δ_{DA}, Δ_{DD} and κ^2 can be obtained from MD simulations. By solving a series of differential equations as in Equation (2), the excited-state populations of chromophores at given Δ_{DA} and Δ_{DD} values can be obtained as a function of time. Finally, η_{FRET} at given Δ_{DA} and Δ_{DD} values can be obtained by averaging Φ_{FRET} of all donors obtained from simulations. To properly reflect the distributions of structural parameters in the simulations, it is desirable to sample more than 100,000 occurrences.

SI Reference

[1] W.-D. Jang, C.-H. Lee, M.-S. Choi, M. Osada, *J. Porphyrins Phthalocyanines*, **2009**, 13, 787-793.

[2] J. Wang, R. M. Wolf, J. W. Caldwell, P. A. Kollman, D. A. Case, *J. Comput. Chem.*, **2004**, 25, 1157–1174.

[3] C. I. Bayly, P. Cieplak, W. Cornell, P. A. Kollman, *J. Phys. Chem.*, **1993**, 97, 10269–10280 (1993).

[4] Y. Shao, Z. Gan, E. Epifanovsky, A. T.B. Gilbert, M. Wormit, J. Kussmann, A. W. Lange, A. Behn, J. Deng, X. Feng, D. Ghosh, M. Goldey, P. R. Horn, L. D. Jacobson, I. Kaliman, R. Z. Khaliullin, T. Kuś, A. Landau, J. Liu, E. I. Proynov, Y. M. Rhee, R. M. Richard, M. A. Rohrdanz, R. P. Steele, E. J. Sundstrom, H. L. Woodcock III, P. M. Zimmerman, D. Zuev, B. Albrecht, E. Alguire, B. Austin, G. J. O. Beran, Y. A. Bernard, E. Berquist, K. Brandhorst, K. B. Bravaya, S. T. Brown, D. Casanova, C.-M. Chang, Y. Chen, S. H. Chien, K. D. Closser, D. L. Crittenden, M. Diedenhofen, R. A. DiStasio Jr., H. Do, A. D. Dutoi, R. G. Edgar, S. Fatehi, L. Fusti-Molnar, A. Ghysels, A. Golubeva-Zadorozhnaya, J. Gomes, M. W. D. Hanson-Heine, P. H. P. Harbach, A. W. Hauser, E. G. Hohenstein, Z. C. Holden, T.-C. Jagau, H. Ji, B. Kaduk, K. Khistyaev, J. Kim, J. Kim, R. A. King, P. Klunzinger, D. Kosenkov, T. Kowalczyk, C. M. Krauter, K. U. Lao, A. D. Laurent, K. V. Lawler, S. V. Levchenko, C. Y. Lin, F. Liu, E. Livshits, R. C. Lochan, A. Luenser, P. Manohar, S. F. Manzer, S.-P. Mao, N. Mardirossian, A. V. Marenich, S. A. Maurer, N. J. Mayhall, E. Neuscamman, C. M. Oana, R. Olivares-Amaya, D. P. O'Neill, J. A. Parkhill, T. M. Perrine, R. Peverati, A. Prociuk, D. R. Rehn, E. Rosta, N. J. Russ, S. M. Sharada, S. Sharma, D. W. Small, A. Sodt, T. Stein, D. Stück, Y.-C. Su, A. J. W. Thom, T. Tsuchimochi, V. Vanovschi, L. Vogt, O. Vydrov, T. Wang, M. A. Watson, J. Wenzel, A. White, C. F. Williams, J. Yang, S. Yeganeh, S. R. Yost, Z.-Q. You, I. Y. Zhang, X. Zhang, Y. Zhao, B. R. Brooks, G. K. L. Chan, D. M. Chipman, C. J. Cramer, W. A. Goddard III, M.

S. Gordon, W. J. Hehre, A. Klamt, H. F. Schaefer III, M. W. Schmidt, C. D. Sherrill, D. G. Truhlar, A. Warshel, X. Xu, A. Aspuru-Guzik, R. Baer, A. T. Bell, N. A. Besley, J.-D. Chai, A. Dreuw, B. D. Dunietz, T.R. Furlani, S. R. Gwaltney, C.-P. Hsu, Y. Jung, J. Kong, D. S. Lambrecht, W. Z. Liang, C. Ochsenfeld, V. A. Rassolov, L. V. Slipchenko, J. E. Subotnik, T. V. Voorhis, J. M. Herbert, A. I. Krylov, P. M.W. Gill, M. Head-Gordon, *Mol. Phys.*, **2015**, 113, 184–215.

[5] G. Bussi, D. Donadio, M. Parrinello, *J. Chem. Phys.*, **2007**, 126, 014101.

[6] M. Parrinello, A. Rahman, *J. Appl. Phys.*, **1981**, 52, 7182–7190.

[7] J. Larsen, B. Brüggemann, T. Polívka, V. Sundström, E. Åkesson, J. Sly, M. J. Crossley, *J. Phys. Chem. A*, **2005**, 109, 10654−10662.

[8] J. Larsen, B. Brüggemann, J. Sly, M. J. Crossley, V. Sundström, E. Åkesson, *Chem. Phys. Lett.*, **2006**, 433, 159-164.

[9] S. Cho, W.-S. Li, M.-C. Yoon, T. K. Ahn, D.-L. Jiang, J. Kim, T. Aida, D. Kim, *Chem. Eur. J.*, **2006**, 12, 7576-7584.

[10] K. K. Jensen, S. B. van Berlekom, J. Kajanus, J. Mårtensson, B. Albinsson, *J. Phys. Chem. A*, **1997**, 101, 2218-2220.

[11] B. Wallace, P. J. Atzberger, *PLoS One*, **2017**, 12, No. e0177122.

[12] M. H. Jacob, R. N. Dsouza, I. Ghosh, A. Norouzy, T. Schwarzlose, W. M. Nau, *J. Phys. Chem. B*, **2013**, 117, 185−198.

[13] J. S. Melinger, A. Khachatrian, M. G. Ancona, S. Buckhout-White, E. R. Goldman, C. M. Spillmann, I. L. Medintz, P. D. Cunningham, *ACS Photonics* **2016**, *3*, 659-669.

[14] L. Olejko, I. Bald, *RSC Adv.*, **2017**, 7, 23924-23934.

[15] S. Buckhout-White, C. M Spillmann, W. R. Algar, A. Khachatrian, J. S. Melinger, E. R. Goldman, M. G. Ancona, I. L. Medintz, *Nat. Commun.* **2014**, *5*, 5615.

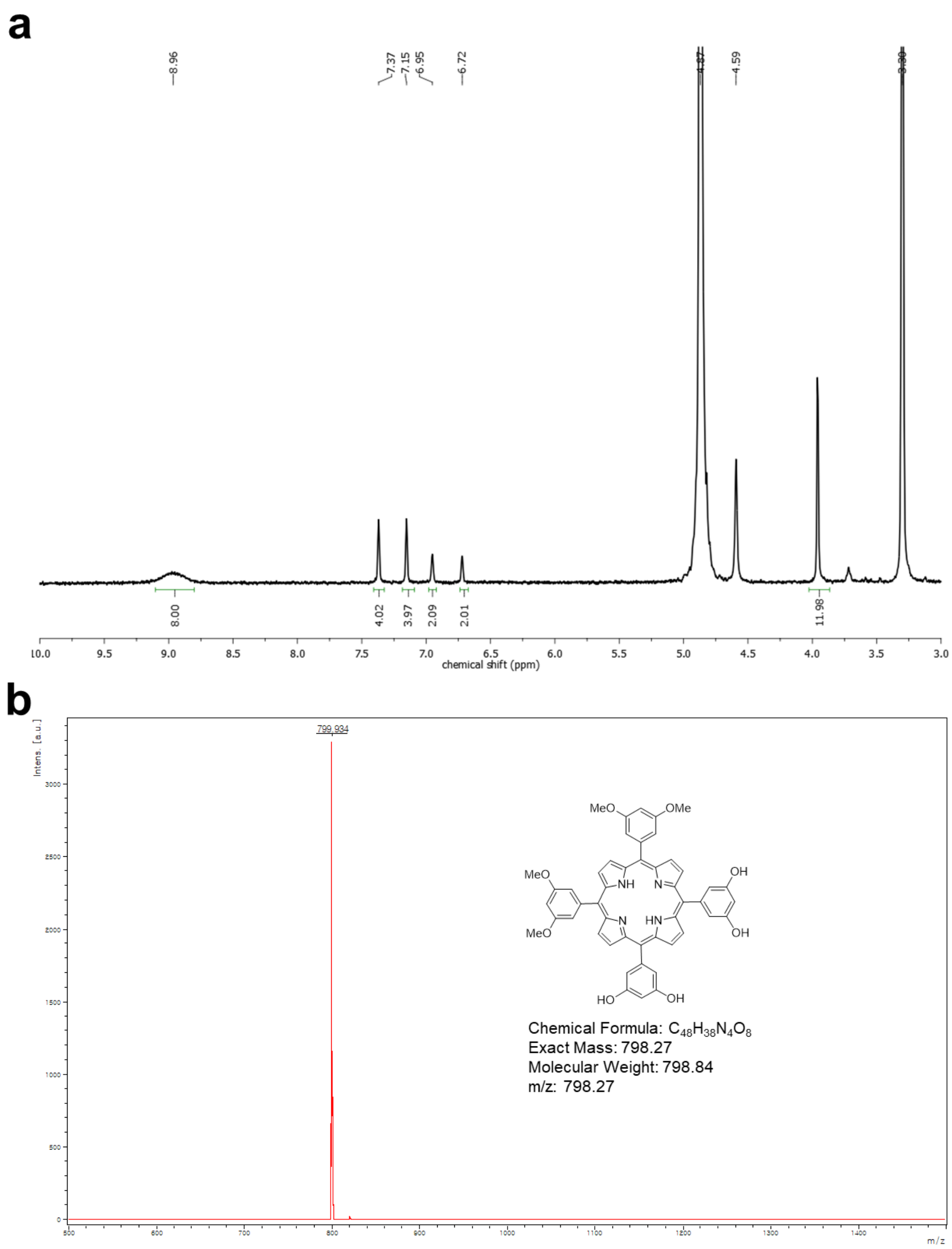

Figure S1. NMR and MALDI TOF MS spectra of *cis*-$(OMe)_4(OH)_4P_{FB}$. (a) ^{1}H-NMR spectrum (400 MHz, MeOD). Residual solvent peaks at 4.87 ppm (water), 3.30 ppm (MeOH) and unknown peaks at 4.59 ppm. (b) MALDI TOF MS spectrum.

a

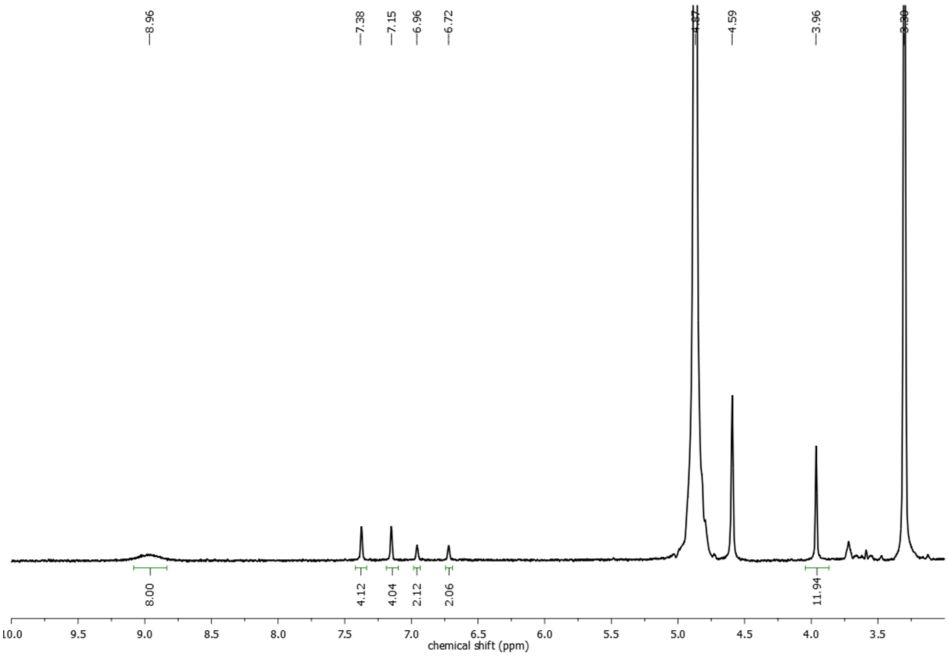

b

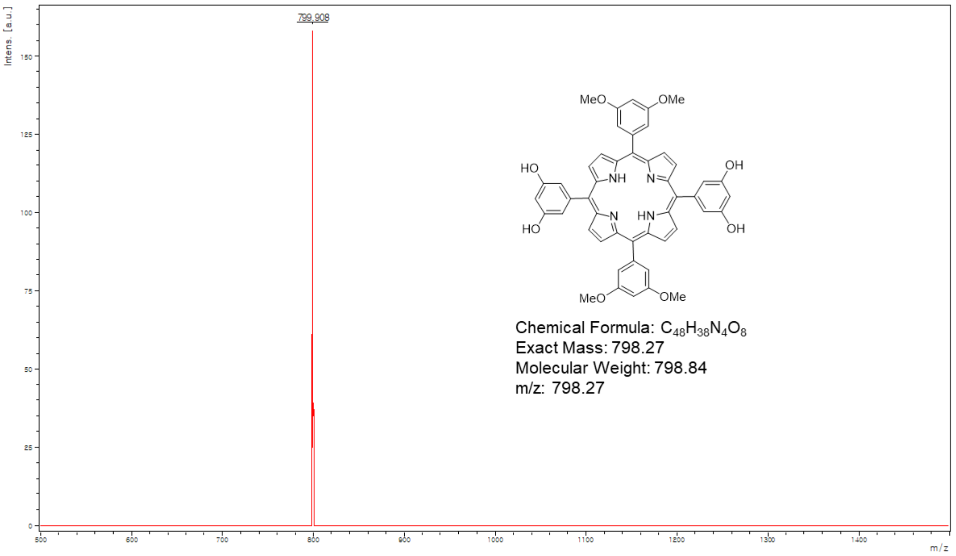

Figure S2. NMR and MALDI TOF MS spectra of *trans*-$(OMe)_4(OH)_4P_{FB}$. (a) ^{1}H-NMR spectrum (400 MHz, MeOD). Residual solvent peaks at 4.87 ppm (water), 3.30 ppm (MeOH) and unknown peaks at 4.59 ppm. (b) MALDI TOF MS spectrum.

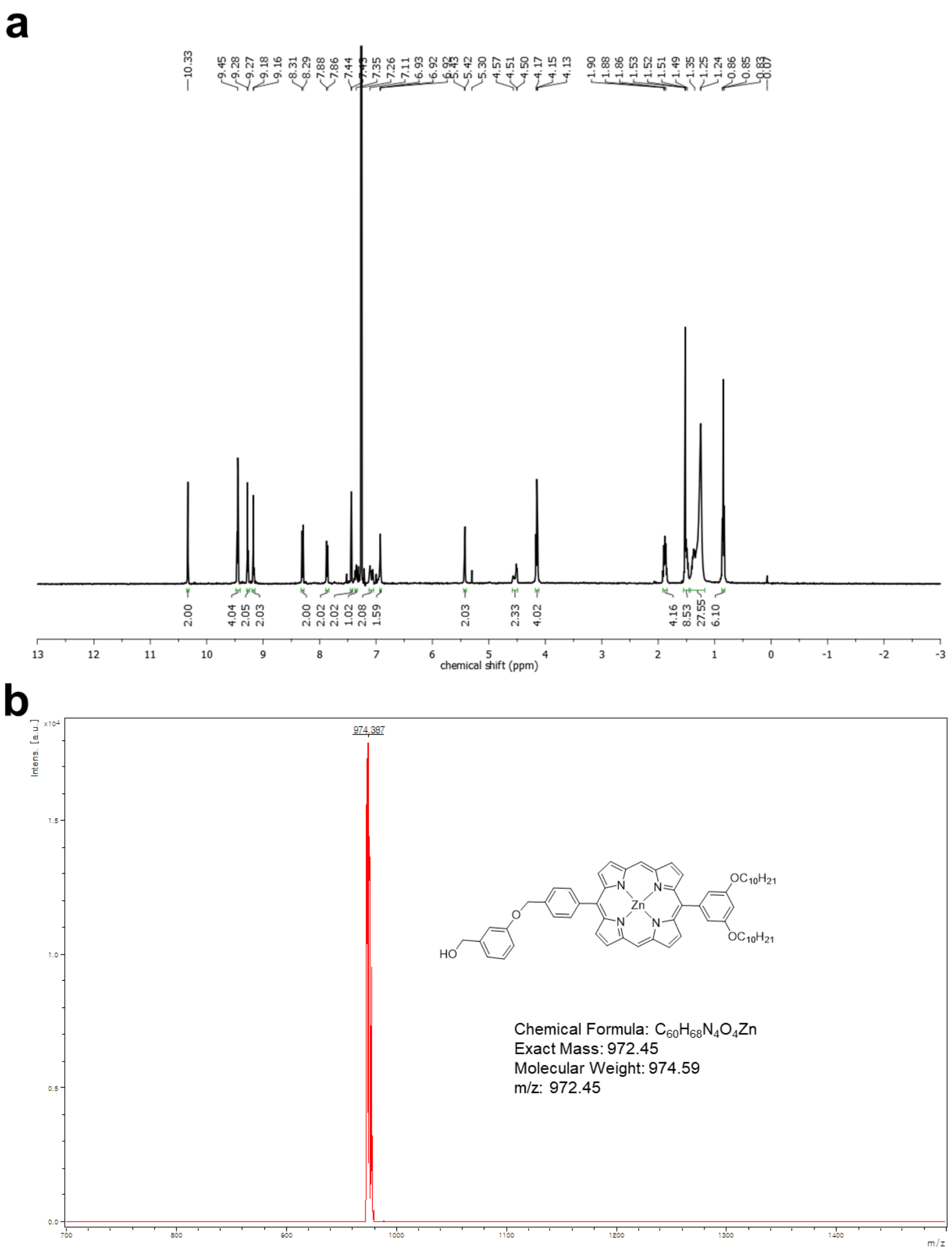

Figure S3. NMR and MALDI TOF MS spectra of P4. (a) ^{1}H-NMR spectrum (400 MHz, $CDCl_3$). Residual solvent peaks at 7.26 ppm ($CHCl_3$), 5.30 ppm (CH_2Cl_2) and 1.53 ppm (water). (b) MALDI TOF MS spectrum.

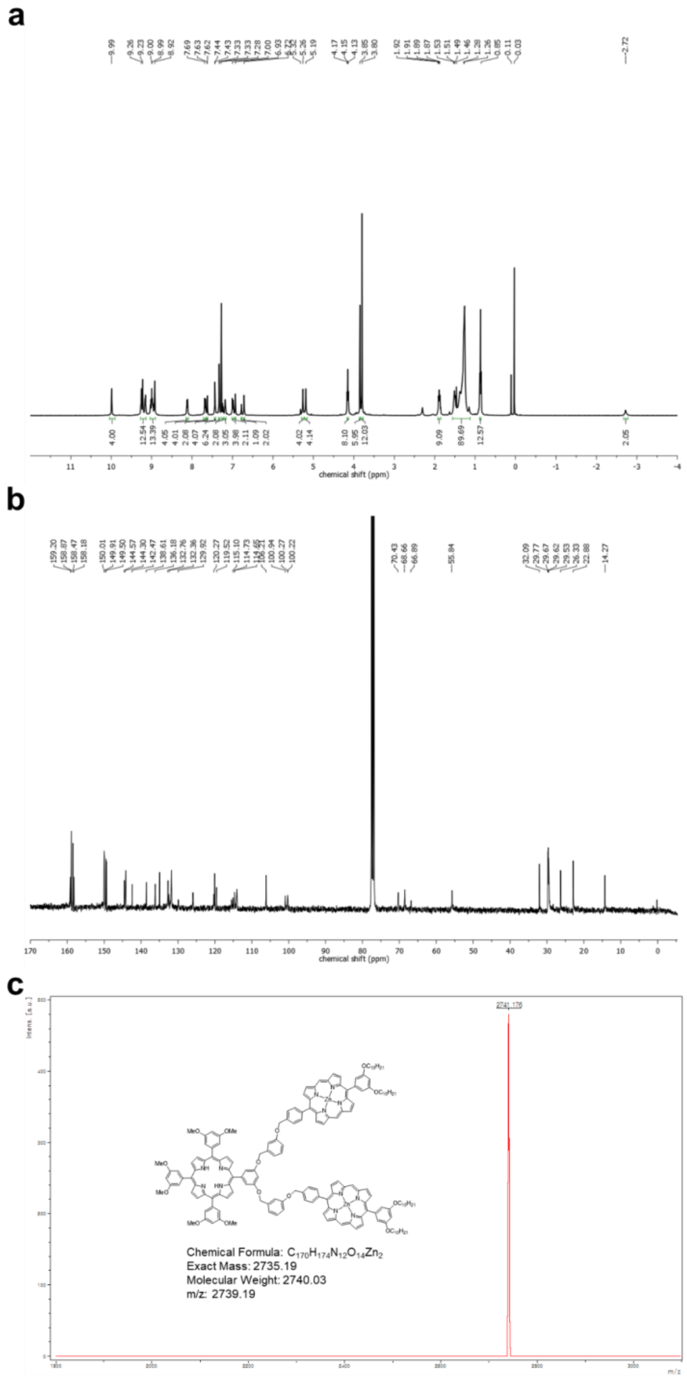

Figure S4. NMR and MALDI TOF MS spectra of MD2A. (a) ^{1}H-NMR spectrum (400 MHz, $CDCl_3$). Residual solvent peaks at 7.28 ppm ($CHCl_3$) and 5.32 ppm (CH_2Cl_2). (b) ^{13}C-NMR spectrum (100 MHz, $CDCl_3$). (c) MALDI TOF MS spectrum.

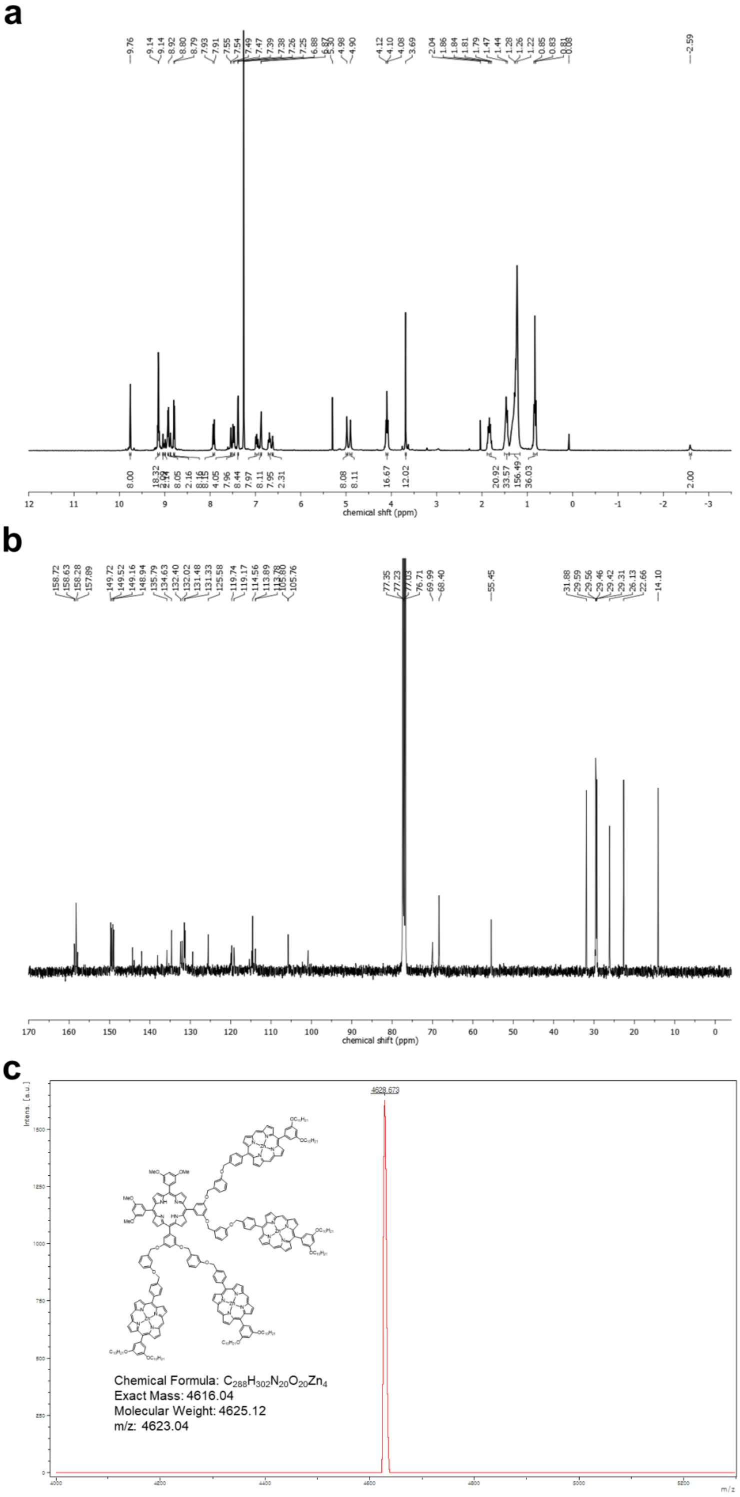

Figure S5. NMR and MALDI TOF MS spectra of MD4A. (a) ^{1}H-NMR spectrum (400 MHz, $CDCl_3$). Residual solvent peaks at 7.26 ppm ($CHCl_3$), 5.30 ppm (CH_2Cl_2) and 1.53 ppm (water). (b) ^{13}C-NMR spectrum (100 MHz, $CDCl_3$). (c) MALDI TOF MS spectrum.

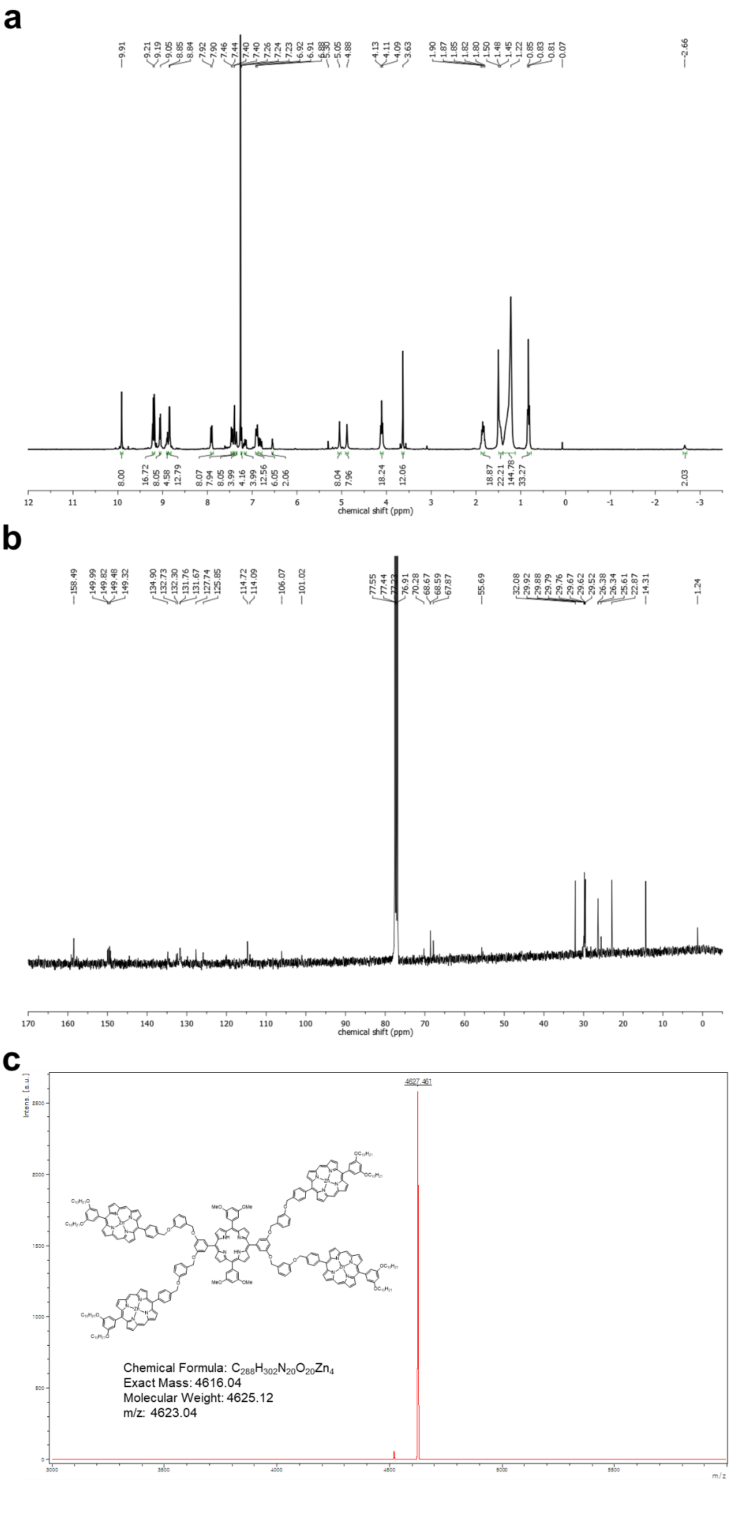

Figure S6. NMR and MALDI TOF MS spectra of LD4A. (a) ^{1}H-NMR spectrum (400 MHz, $CDCl_3$). (b) ^{13}C-NMR spectrum (100 MHz, $CDCl_3$). (c) MALDI TOF MS spectrum.

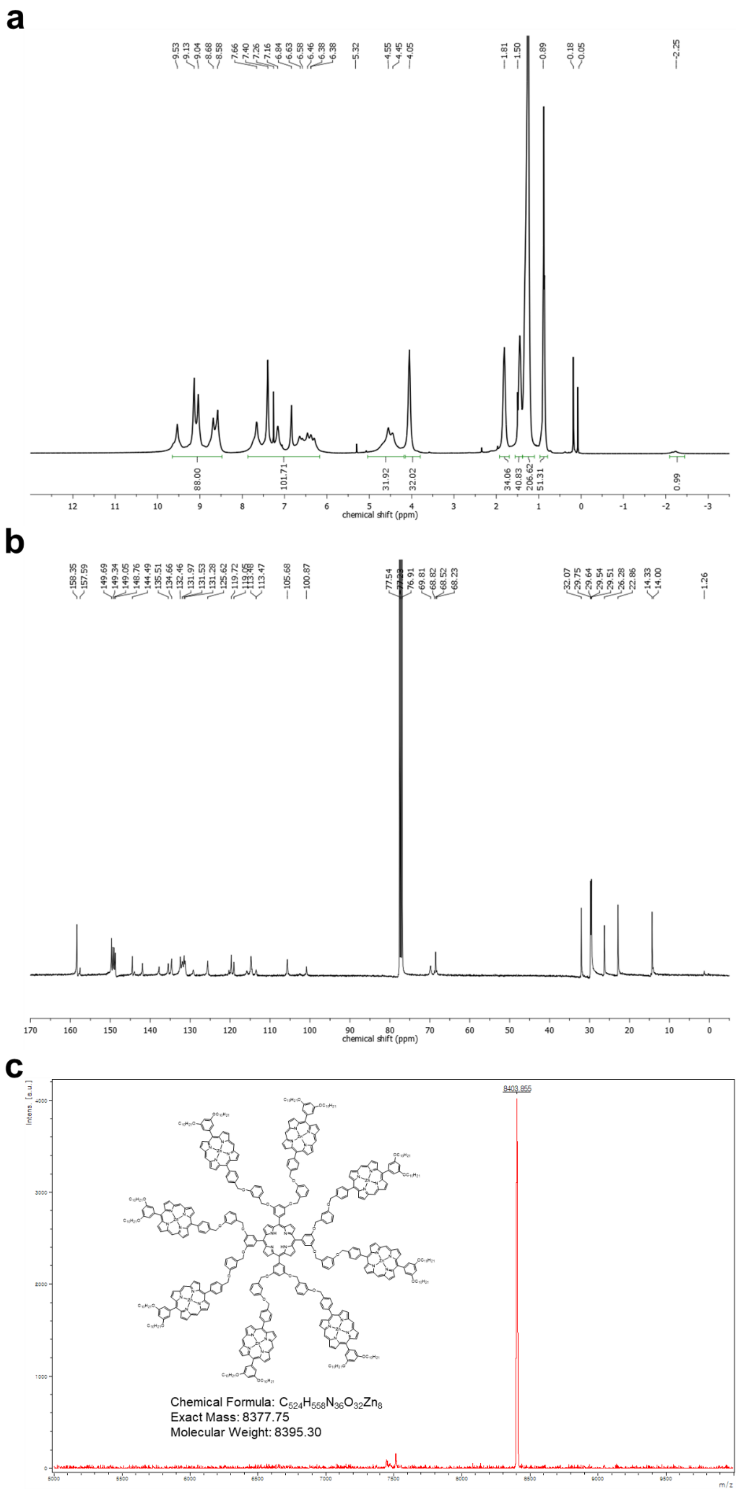

Figure S7. NMR and MALDI TOF MS spectra of MD8A. (a) ^{1}H-NMR spectrum (400 MHz, $CDCl_3$). Residual solvent peaks at 7.26 ppm ($CHCl_3$) and 5.32 ppm (CH_2Cl_2). (b) ^{13}C-NMR spectrum (100 MHz, $CDCl_3$). (c) MALDI TOF MS spectrum.

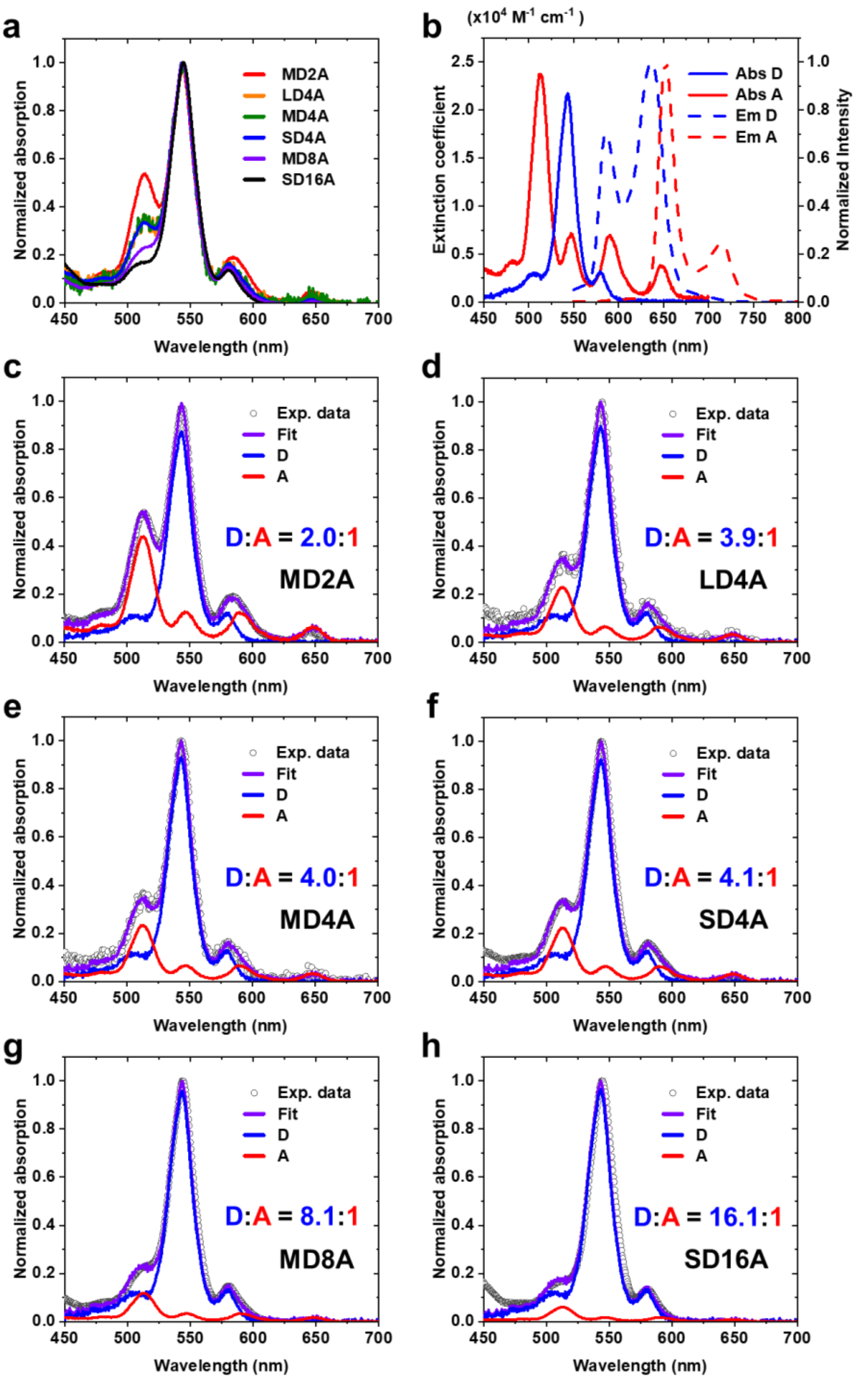

Figure S8. Steady-state absorption and emission spectra of monomeric porphyrins and multi-porphyrin dendrimers. (a) Normalized electronic absorption spectra of multi-porphyrin dendrimers. (b) The absorption (solid) and emission spectra (dashed) of the donor (blue) and the acceptor (red). (c - h) Fits (purple) of the experimental absorption spectra of dendrimers

(black circle) by the linear combination of the spectra of the donor (blue) and acceptor (red). The ratio of donor to acceptor in the linear combination fit is shown in each figure.

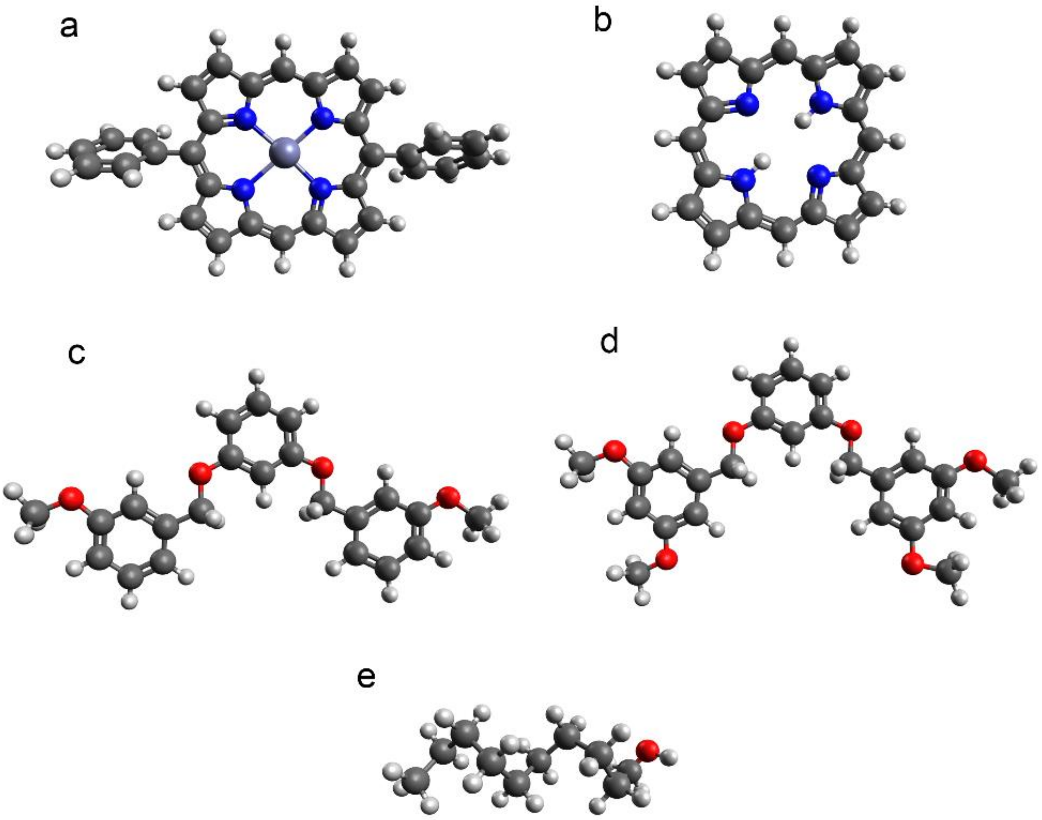

Figure S9. The dendrimer fragments used for fitting the atomic partial charges. (a) The donor fragment. (b) The acceptor fragment. (c) The bridge fragment for MD2A, MD4A, LD4A and MD8A. (d) The bridge fragment for SD4A and SD16A. (e) The hydrocarbon tail attached to the donor.

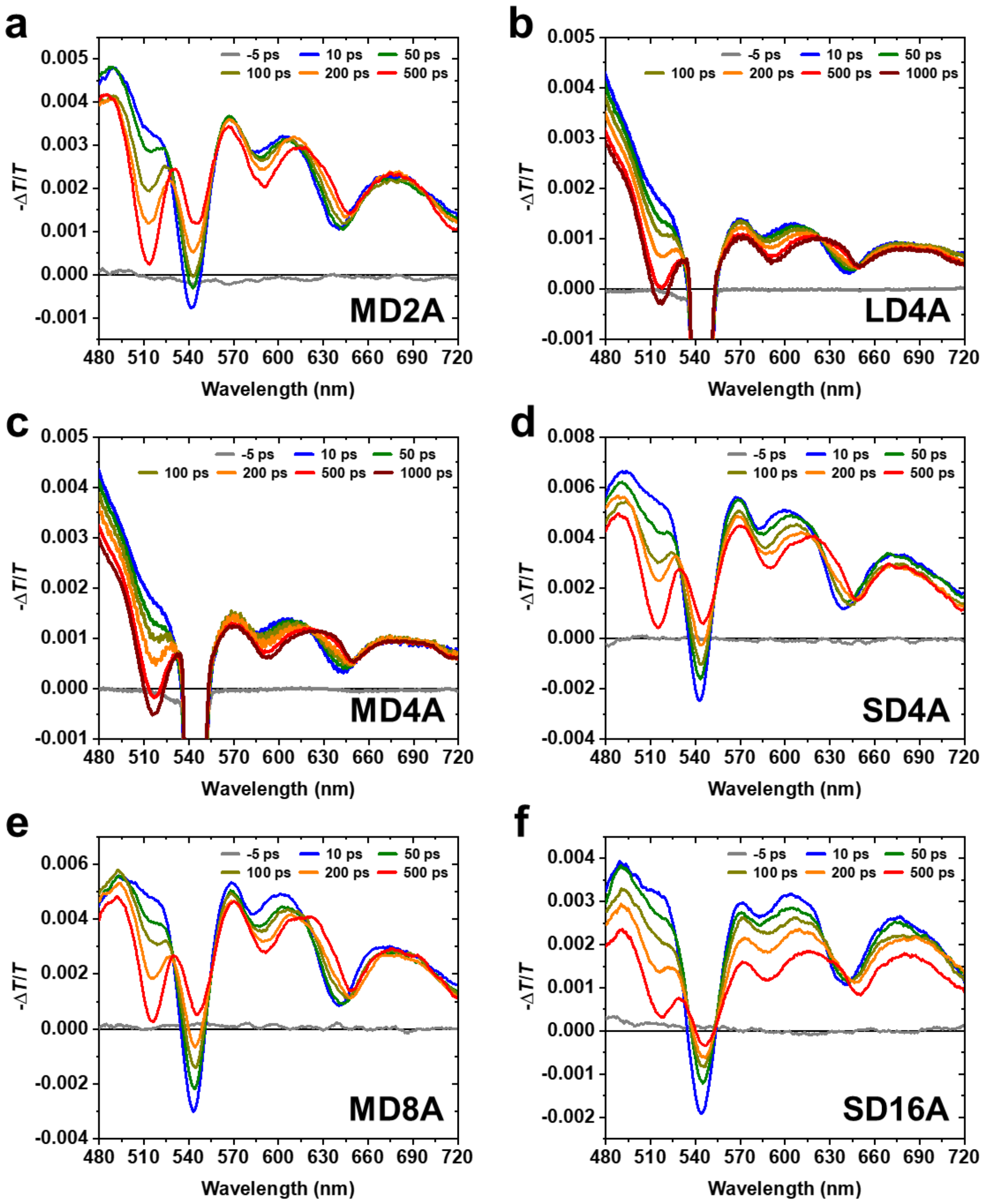

Figure S10. TA spectra of (a) MD2A, (b) LD4A, (c) MD4A, (d) SD4A, (e) MD8A, and (f) SD16A at −5 ps (gray), 10 ps (blue), 50 ps (green), 100 ps (dark yellow), 200 ps (orange), 500 ps (red), and 1000 ps (brown). All TA spectra were measured with the pump wavelength of 540 nm and the pulse energy set to 50 nJ. For LD4A and MD4A, the negative peak is cropped because it is contaminated by the intense scattering by pump pulse.

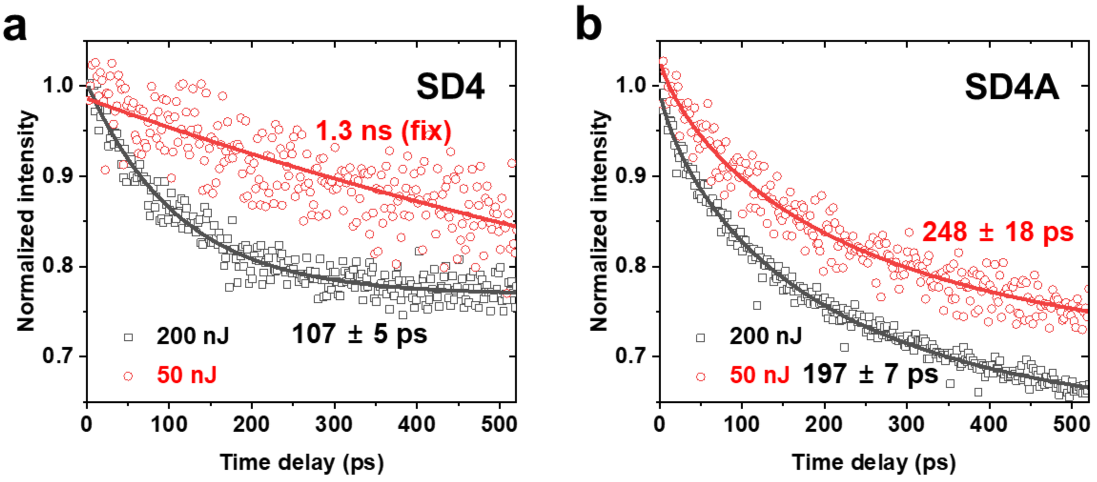

Figure S11. Dependence of the of TA signals on the pulse energy of pump pulses for (a) SD4, and (b) SD4A. The temporal profiles of the TA spectra measured with the pump pulse energies of 50 nJ (black) and 200 nJ (red) are represented by the first right singular vectors obtained from the singular value decomposition of the TA spectra.

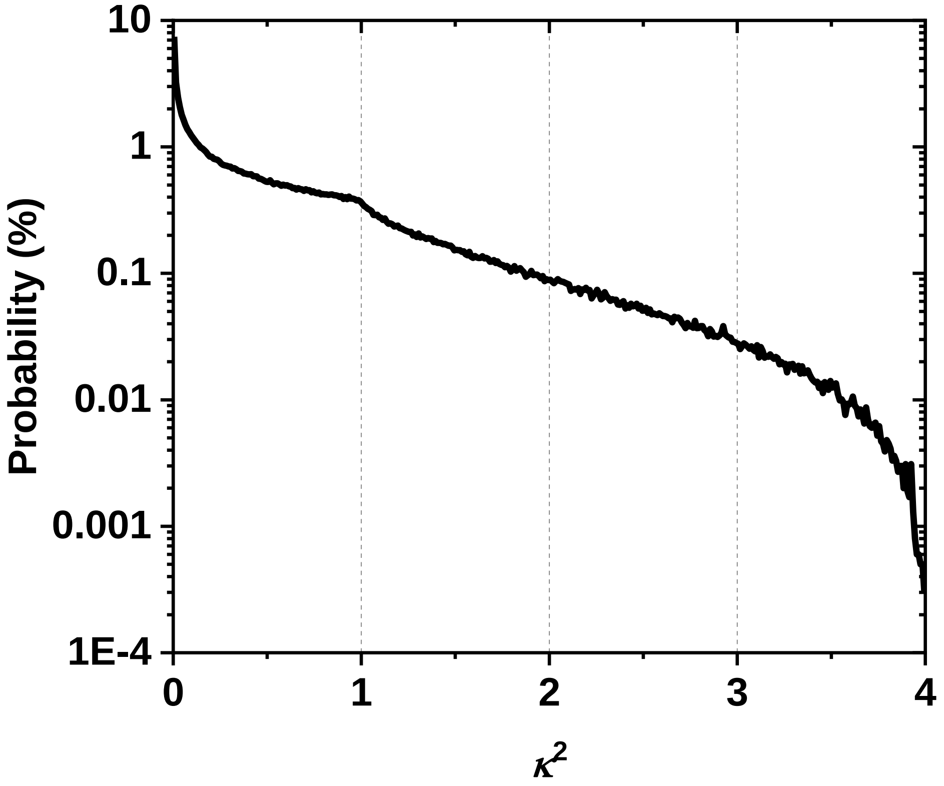

Figure S12. Distribution of orientation factors (κ^2) in static isotropic limit obtained from Monte Carlo simulations. The average value of the distribution is ⅔, which is used for the dynamic isotropic limit.

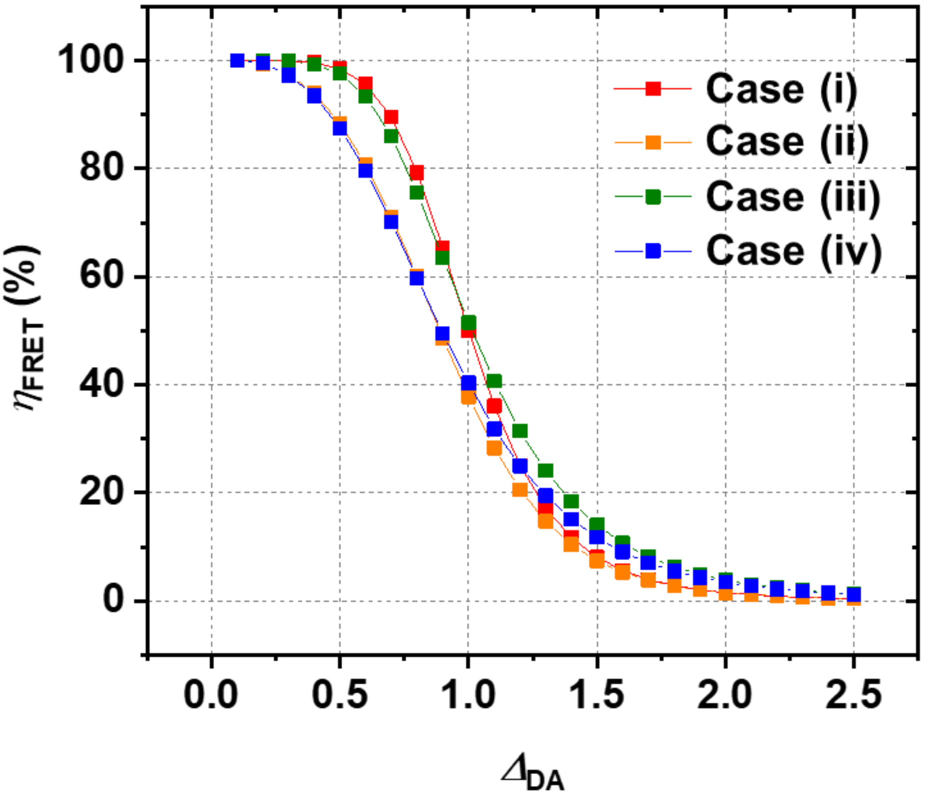

Figure S13. η_{FRET} of the D1A model as a function of Δ_{DA} in cases (i) (blue), (ii) (cyan), (iii) (red), and (iv) (magenta). η_{FRET} was calculated from simulations with given values of Δ_{DA} and κ^2.

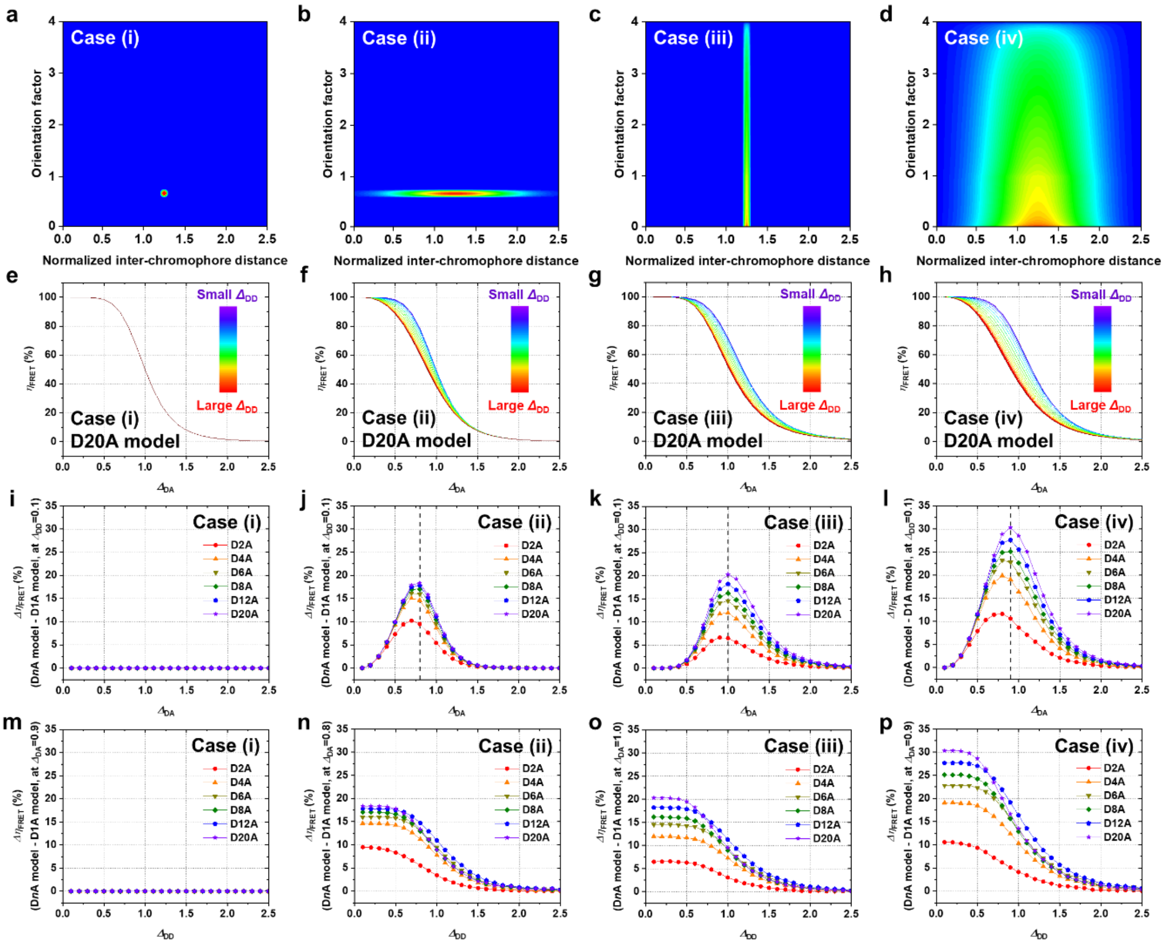

Figure S14. Simulation for calculating η_{FRET} in model MCSs for the four cases. (a - d) Schematic of distributions of the inter-chromophore distances and the orientation factor in the four cases used in our simulations. The distributions are shown in the form of heatmaps where blue and red indicate the lowest and highest values. (e - h) The FRET efficiency (η_{FRET}) of D20A as a function of Δ_{DA} for various Δ_{DD} values. η_{FRET} was calculated from simulations with given values of Δ_{DA}, Δ_{DD}, and κ^2. η_{FRET} curves for various Δ_{DD} values are plotted following a rainbow color scheme, from red (the largest Δ_{DD}) to purple (the smallest Δ_{DD}). (i - l) $\Delta\eta_{FRET}$ of D*n*A relative to D1A, that is η_{FRET}(D*n*A) - η_{FRET}(D1A), as a function of Δ_{DA} with the shortest Δ_{DD} (= 0.1), for n = 2 (red), 4 (orange), 6 (dark yellow), 8 (green), 12 (blue), and 20 (violet) models. The variation curve of $\Delta\eta_{FRET}$ with respect to Δ_{DA} was obtained by

subtracting the η_{FRET} curve of D1A shown in Figure S13 from the η_{FRET} curve of DnA with Δ_{DD} = 0.1. (m - p) $\Delta\eta_{FRET}$ of DnA relative to D1A, that is η_{FRET}(DnA) - η_{FRET}(D1A), as a function of Δ_{DD} with the Δ_{DA} value giving the maximum $\Delta\eta_{FRET}$ for D20A. The variation curve of $\Delta\eta_{FRET}$ with respect to Δ_{DD} was obtained by subtracting the η_{FRET} of D1A (65.3 % for case (i), 60.1 % for case (ii), 51.5 % for case (iii), and 49.5 % for case (iv)) at the Δ_{DA} value giving the maximum $\Delta\eta_{FRET}$ for D20A from η_{FRET} curve of DnA at the same Δ_{DA}.

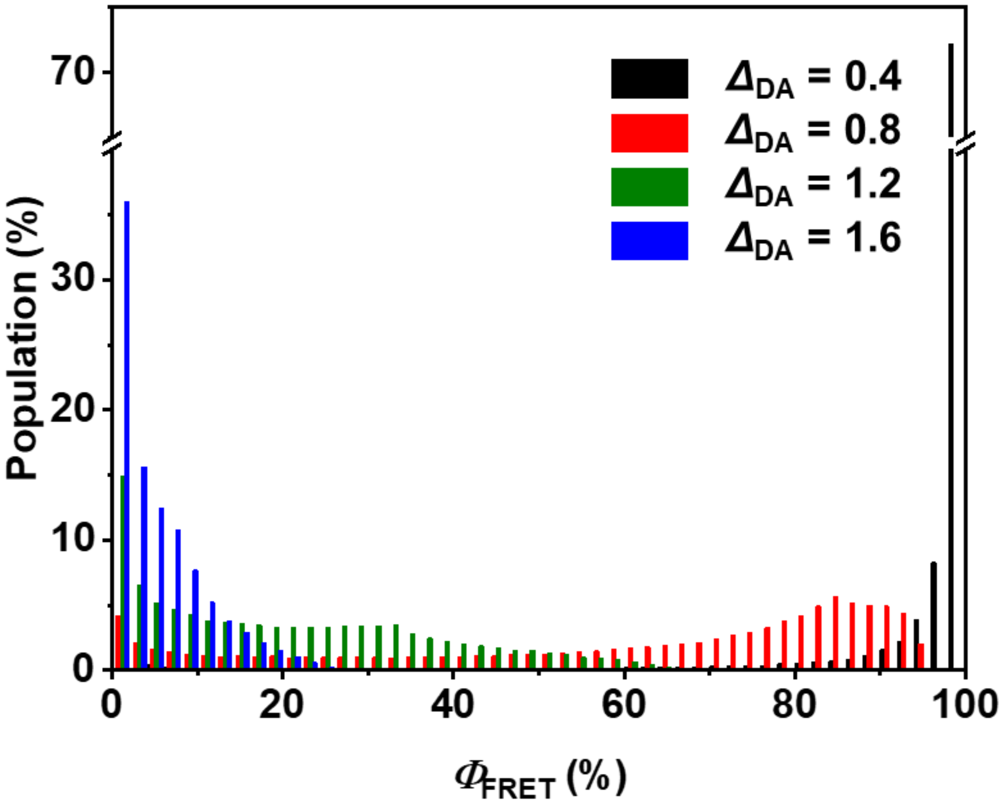

Figure S15. Distribution of Φ_{FRET} in case (ii) at Δ_{DA} = 0.4 (red), 0.8 (green), 1.2 (blue), and 1.6 (cyan).

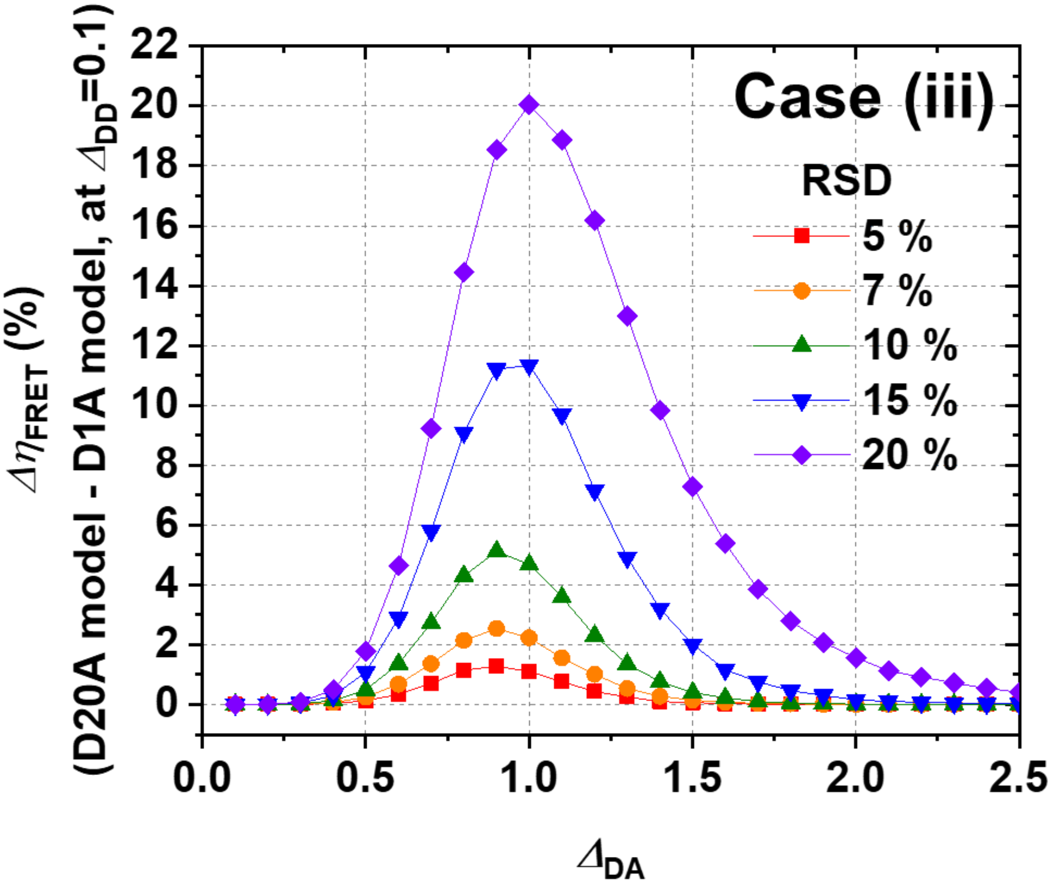

Figure S16. $\Delta\eta_{FRET}$ of D20A relative to D1A, that is η_{FRET}(D20A) - η_{FRET}(D1A), as a function of Δ_{DA} with the shortest Δ_{DD} (= 0.1) and the relative standard deviation of Δ_{DA} (RSD) = 5 % (red), 7 % (orange), 10 % (green), 15 % (blue), and 20 % (purple) in case (iii). The variation curve of $\Delta\eta_{FRET}$ with respect to Δ_{DA} was obtained by subtracting the η_{FRET} curve of D1A shown in Figure S13 from the η_{FRET} curve of D20A with Δ_{DD} = 0.1.

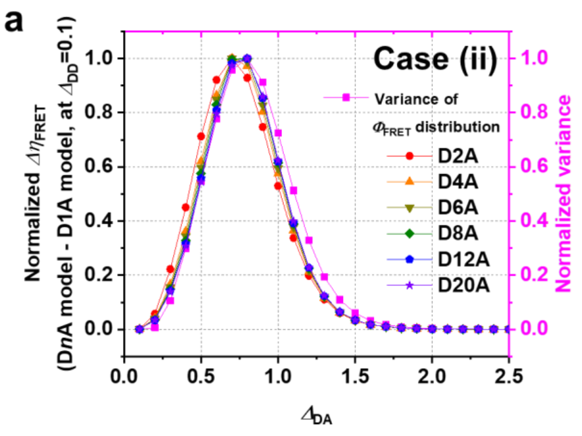

Figure S17. Comparison of the width (quantified by variance) of the distribution of Φ_{FRET} (magenta) and the normalized $\Delta\eta_{\mathrm{FRET}}$ of D2A (red), D4A (orange), D6A (dark yellow), D8A (green), D12A (blue), and D20A (purple) relative to D1A for (a) case (ii) and (b) case (iii). It can be seen that $\Delta\eta_{\mathrm{FRET}}$ and the variance of the distribution of Φ_{FRET} are in good agreement with each other, indicating that the $\Delta\eta_{\mathrm{FRET}}$ value at a given Δ_{DA} is governed by how broad the distribution of Φ_{FRET} is.

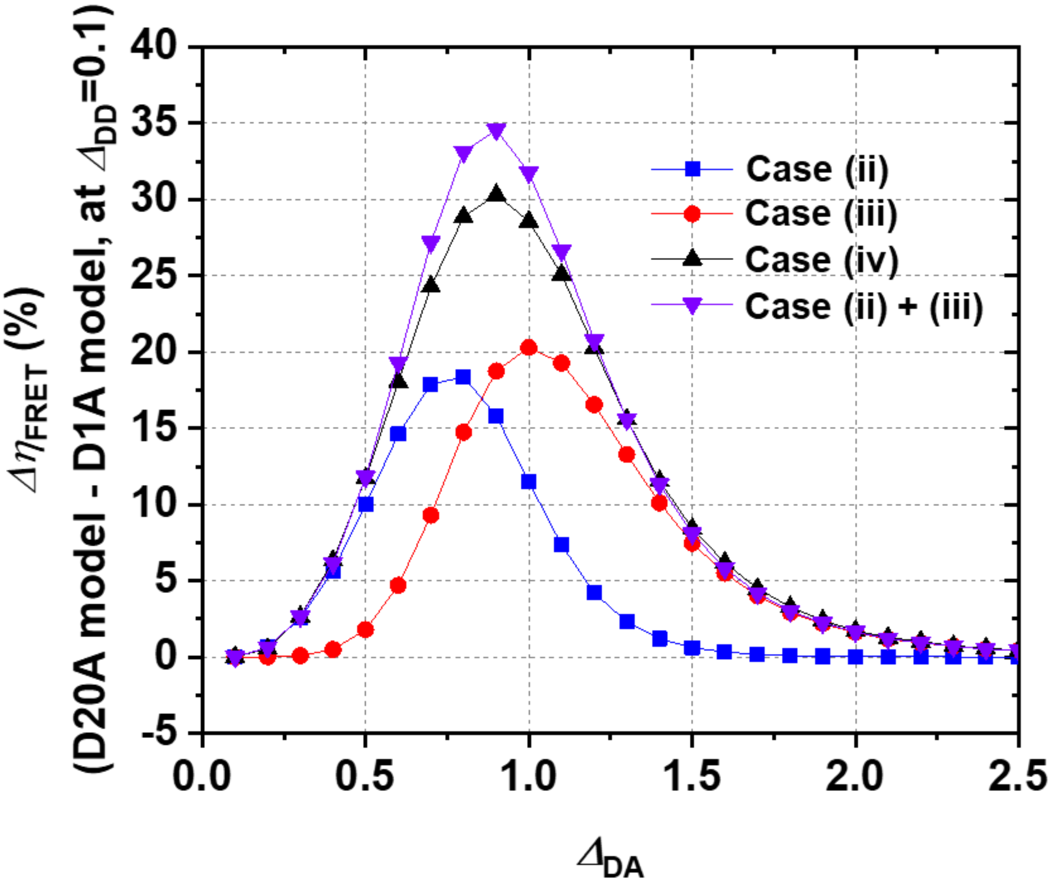

Figure S18. $\Delta\eta_{FRET}$ of D20A relative to D1A, that is η_{FRET}(D20A) - η_{FRET}(D1A), as a function of Δ_{DA} with the shortest Δ_{DD} (= 0.1), for case (ii) (blue), case (iii) (red), and case (iv) (black). The variation curve of $\Delta\eta_{FRET}$ with respect to Δ_{DA} was obtained by subtracting the η_{FRET} curve of D1A shown in Figure S13 from the η_{FRET} curve of D20A with Δ_{DD} = 0.1. Sum of the $\Delta\eta_{FRET}$ curves for case (ii) and (iii) is also shown (purple).

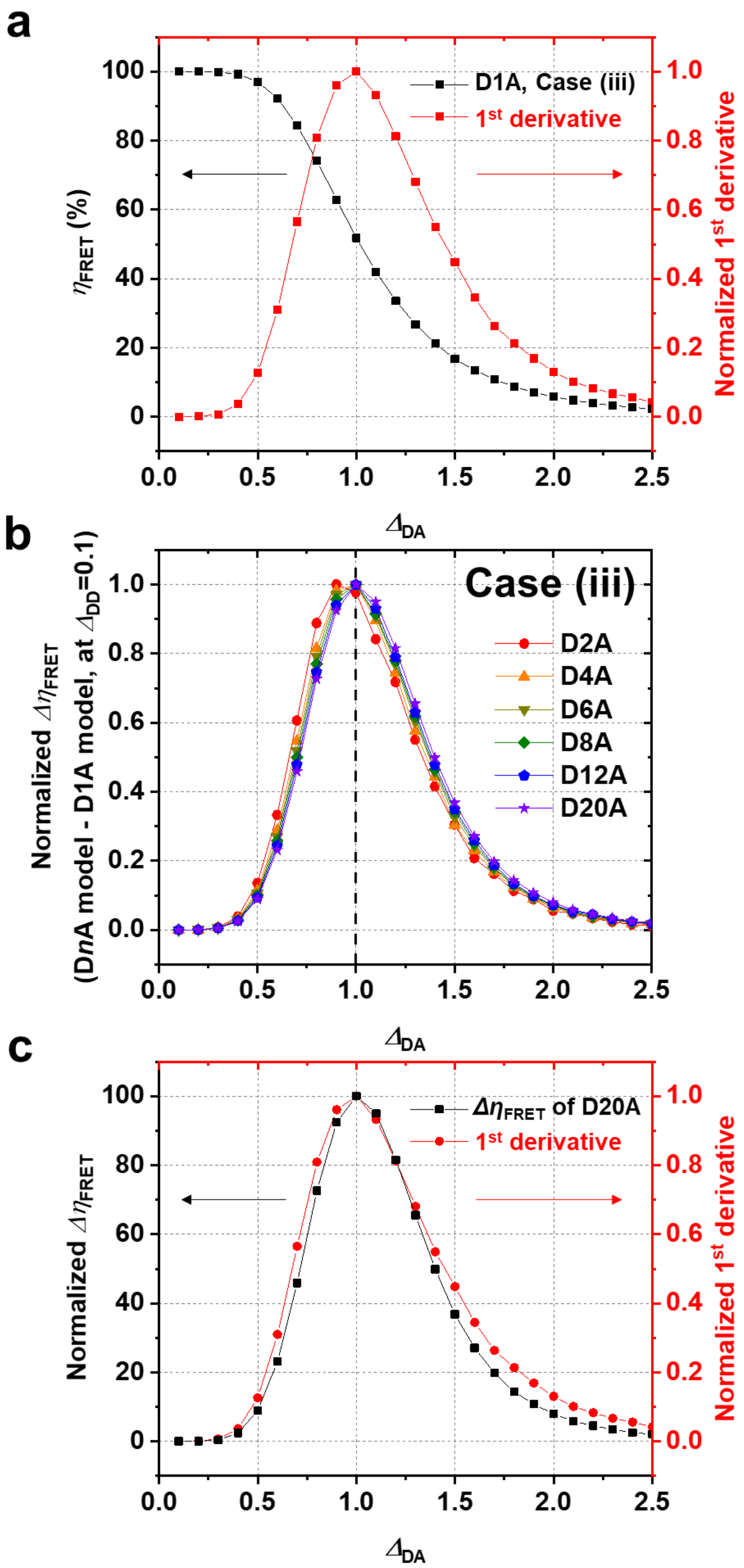

Figure S19. (a) Δ_{DA} dependence of η_{FRET} (black) and the inverse of the normalized 1st derivative of η_{FRET} with respect to Δ_{DA}, $-d\eta_{FRET} / d\Delta_{DA}$ (red), of D1A. Δ_{DD} was set to be 0.1.

(b) Δ_{DA} dependences of the normalized $\Delta\eta_{FRET}$'s of D2A (red), D4A (orange), D6A (dark yellow), D8A (green), D12A (blue), and D20A (purple) relative to D1A. Δ_{DD} was set to be 0.1. It can be seen that the normalized $\Delta\eta_{FRET}$'s of all the model MCSs have similar shapes to each other. (c) Comparison of the normalized $\Delta\eta_{FRET}$ of D20A (black) and $-\mathrm{d}\eta_{FRET}$ / $\mathrm{d}\Delta_{DA}$ of D1A (red). It can be seen that $\Delta\eta_{FRET}$ of D20A and $-\mathrm{d}\eta_{FRET}$ / $\mathrm{d}\Delta_{DA}$ of D1A are in good agreement with each other, indicating that the $\Delta\eta_{FRET}$ value at a given Δ_{DA} is governed by the slope of η_{FRET} at the corresponding Δ_{DA}. We note that $\mathrm{d}\eta_{FRET}$ / $\mathrm{d}\Delta_{DA}$ of D1A was used for comparison with $\Delta\eta_{FRET}$ of D20A to eliminate any effect of homo-FRET on η_{FRET}.

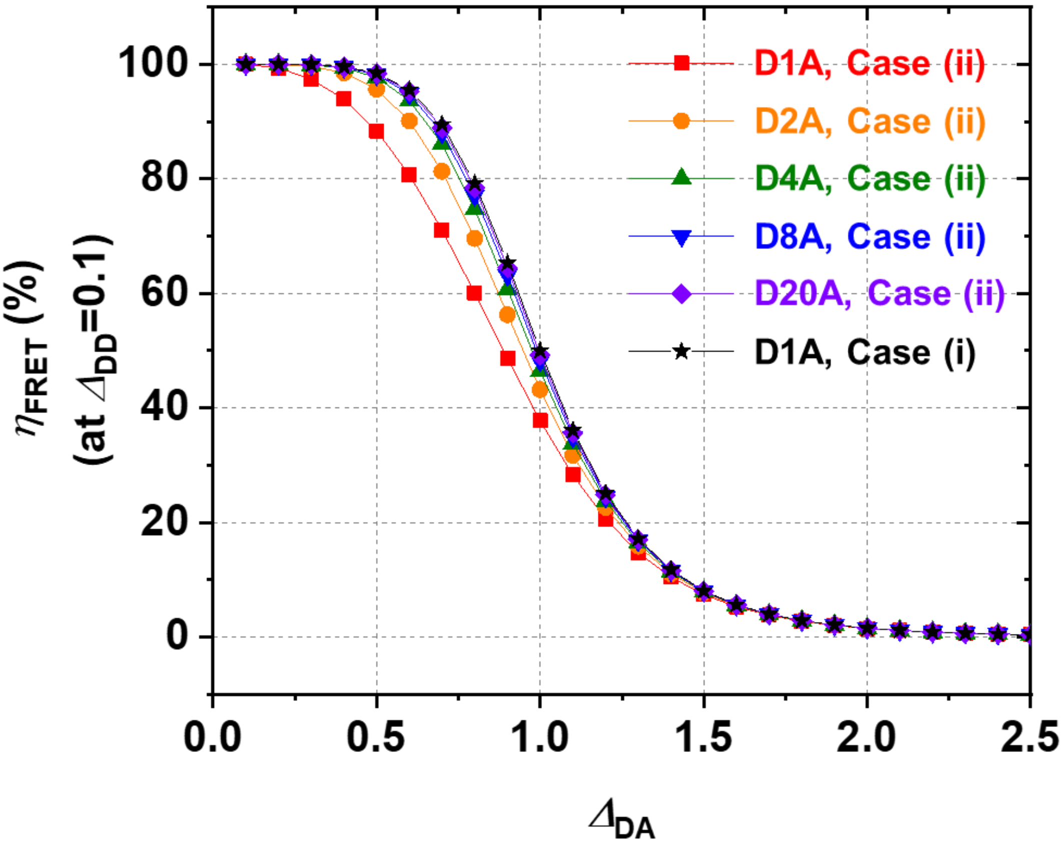

Figure S20. η_{FRET} of D1A (red), D2A (orange), D4A (green), D8A (blue), D20A (purple) in case (ii), and η_{FRET} of D1A (black) in case (i) as a function of Δ_{DA} at Δ_{DD} = 0.1. As n_{D} increases, the η_{FRET} curve of D*n*A in case (ii) converges the η_{FRET} curve of D1A in case (i).

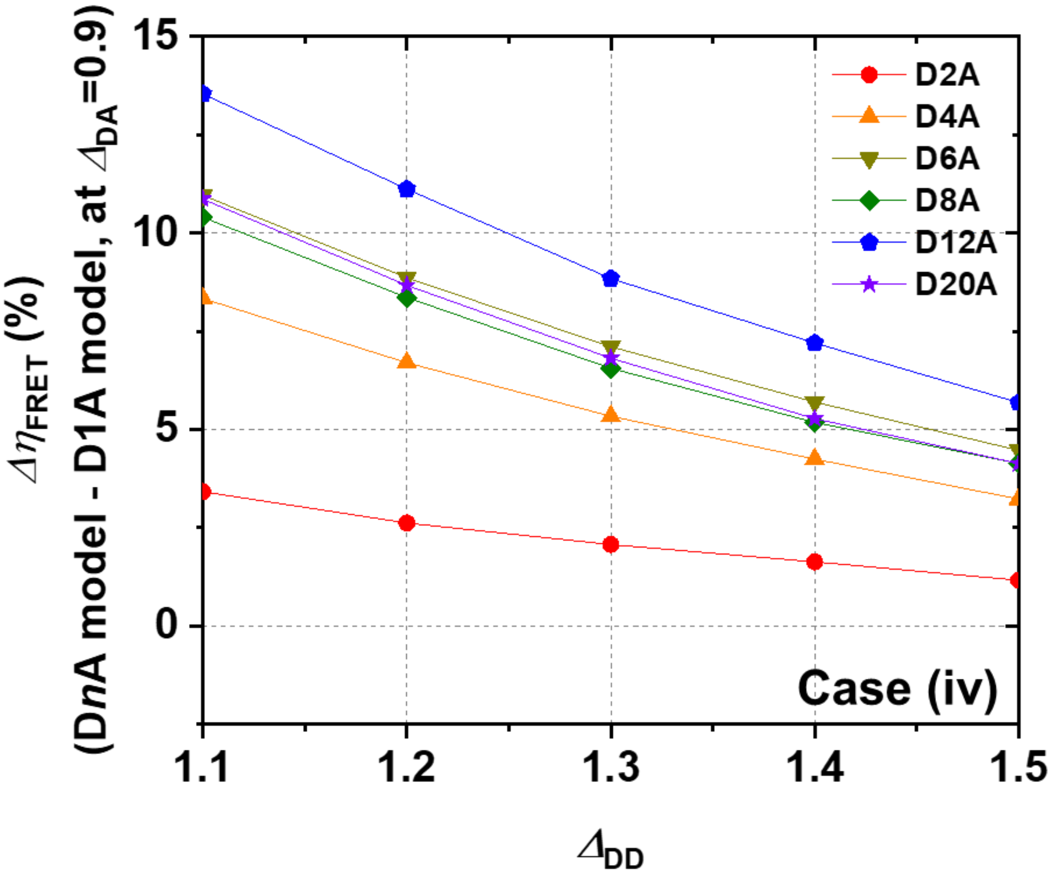

Figure S21. $\Delta\eta_{FRET}$ of D*n*A relative to D1A in case (iv), that is η_{FRET}(D*n*A) - η_{FRET}(D1A), as a function of Δ_{DD} with Δ_{DA} = 0.9. The variation curve of $\Delta\eta_{FRET}$ with respect to Δ_{DD} was obtained by subtracting η_{FRET} of D1A at Δ_{DA} = 0.9 (49.5 %) from the η_{FRET} curve of D*n*A at the same Δ_{DA}.

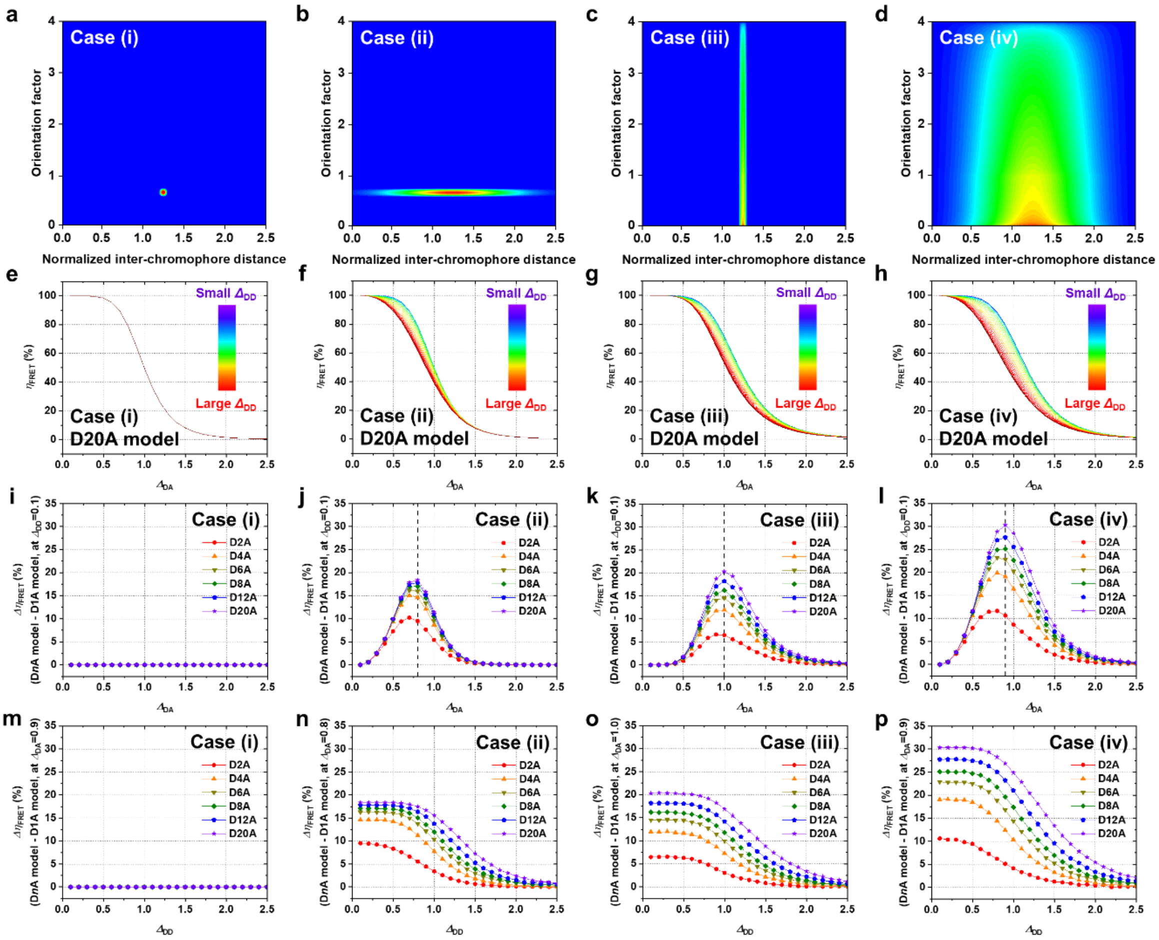

Figure S22. Simulation for calculating η_{FRET} in model MCSs with n_{ND} set to be equal to n_D. (a - d) Schematic of distributions of the inter-chromophore distances and the orientation factor in the four cases used in our simulations. The distributions are shown in the form of heatmaps where blue and red indicate the lowest and highest values. (e - h) η_{FRET} of D20A as a function of Δ_{DA} with various Δ_{DD} values. η_{FRET} was calculated from simulations with given values of Δ_{DA}, Δ_{DD}, and κ^2. η_{FRET} curves for various Δ_{DD} values are plotted following a rainbow color scheme, from red (the largest Δ_{DD}) to purple (the smallest Δ_{DD}). (i - l) $\Delta\eta_{FRET}$ of D*n*A relative to D1A, that is η_{FRET}(D*n*A) - η_{FRET}(D1A), as a function of Δ_{DA} with the shortest Δ_{DD} (= 0.1), for n = 2 (red), 4 (orange), 6 (dark yellow), 8 (green), 12 (blue), and 20 (violet) models. The variation curve of $\Delta\eta_{FRET}$ with respect to Δ_{DA} was obtained by subtracting the η_{FRET} curve of

D1A shown in Figure S13 from the η_{FRET} curve of DnA with Δ_{DD} = 0.1. (m - p) $\Delta\eta_{\mathrm{FRET}}$ of DnA and D1A, that is η_{FRET}(DnA) - η_{FRET}(D1A), as a function of Δ_{DD} with the Δ_{DA} value giving the maximum $\Delta\eta_{\mathrm{FRET}}$ for D20A. The variation curve of $\Delta\eta_{\mathrm{FRET}}$ with respect to Δ_{DD} was obtained by subtracting the η_{FRET} of D1A (65.3 % for case (i), 60.1 % for case (ii), 51.5 % for case (iii), and 49.5 % for case (iv)) at the Δ_{DA} value giving the maximum $\Delta\eta_{\mathrm{FRET}}$ for D20A from η_{FRET} curve of DnA at the same Δ_{DA}. We note that figures in second and fourth columns differ from the figures in Figure S14 in the same positions.
